# Implications of a short carbon pulse on biofilm formation on mica schist in microcosms with deep crystalline bedrock groundwater

**DOI:** 10.3389/fmicb.2023.1054084

**Published:** 2023-02-02

**Authors:** Maija Nuppunen-Puputti, Riikka Kietäväinen, Ilmo Kukkonen, Malin Bomberg

**Affiliations:** ^1^VTT Technical Research Centre of Finland Ltd., Espoo, Finland; ^2^Geological Survey of Finland, Espoo, Finland; ^3^Department of Physics, University of Helsinki, Helsinki, Finland

**Keywords:** Outokumpu deep drill hole, ICDP, acetate, methane, methanol, SRB, fungi, Fennoscandian shield

## Abstract

Microbial life in the deep subsurface occupies rock surfaces as attached communities and biofilms. Previously, epilithic Fennoscandian deep subsurface bacterial communities were shown to host genetic potential, especially for heterotrophy and sulfur cycling. Acetate, methane, and methanol link multiple biogeochemical pathways and thus represent an important carbon and energy source for microorganisms in the deep subsurface. In this study, we examined further how a short pulse of low-molecular-weight carbon compounds impacts the formation and structure of sessile microbial communities on mica schist surfaces over an incubation period of ∼3.5 years in microcosms containing deep subsurface groundwater from the depth of 500 m, from Outokumpu, Finland. The marker gene copy counts in the water and rock phases were estimated with qPCR, which showed that bacteria dominated the mica schist communities with a relatively high proportion of epilithic sulfate-reducing bacteria in all microcosms. The dominant bacterial phyla in the microcosms were Proteobacteria, Firmicutes, and Actinobacteria, whereas most fungal genera belonged to Ascomycota and Basidiomycota. Dissimilarities between planktic and sessile rock surface microbial communities were observed, and the supplied carbon substrates led to variations in the bacterial community composition.

## 1. Introduction

Microbial life on Earth extends to several kilometers depths into the subsurface below the continents and the ocean floor forming the deep biosphere. These intra-terrestrial microbial communities exist as attached epilithic and endolithic biofilm communities or as planktic communities in the deep groundwater (Onstott, 1997; Pedersen, 1997, 2000; Onstott et al., 2003; Itävaara et al., 2011; Edwards et al., 2012; Colwell and D’Hondt, 2013; Escudero et al., 2018b; Smith et al., 2019; Casar et al., 2020; Kraus, 2021; Nuppunen-Puputti et al., 2021). The deep continental subsurface contains a great proportion of Earth’s microbial biomass, with ∼20–80% of the microbial cells living attached on surfaces as single cells and as microcolonies or in biofilms (McMahon and Parnell, 2014; Bar-On et al., 2018; Magnabosco et al., 2018; Bar-On and Milo, 2019; Flemming and Wuertz, 2019). As the attached rock surface microbial communities have been shown to diverge from the planktic communities at many continental subsurface locations (Momper et al., 2017; Kraus, 2021; Nuppunen-Puputti et al., 2021), the metabolic traits of microbial communities likely differ in the rock surface from the planktic ones. There is a clear knowledge gap on how direct interaction with mineral surfaces supports microbial community functionality in deep bedrock environments. As biofilm communities form a major proportion of the deep continental subsurface microbiome, there is a need to elucidate the carbon cycling in the rock-hosted communities. This will upgrade the understanding of overall metabolic feedback systems supporting the deep subsurface microbial life.

The role of carbon compounds in the microbial attachment processes in the deep biosphere is poorly known. Organic carbon compounds, such as polysaccharides, are part of the conditioning film forming over rock surfaces in the deep groundwater allowing the microbes to begin their attachment process (Schäfer et al., 2015). In addition to the effects on the surface charge properties, small 1-carbon (C1) and 2-carbon (C2) compounds have a significant role as an energy and carbon source in the biogeochemical cycles in the deep oligotrophic bedrock biosphere (Pedersen, 2012; Rajala et al., 2015; Bomberg et al., 2017; Purkamo et al., 2017; Rajala and Bomberg, 2017; Escudero et al., 2018a; Miettinen et al., 2018; Nuppunen-Puputti et al., 2018; Sahu et al., 2022). The dissolved organic carbon (DOC) pool can include various organic compounds such as methanol and acetate. These small carbon compounds link diverse metabolic pathways that are used by, e.g., methylotrophs, methanotrophs, acetotrophs, acetogens, and methanogens (Kotelnikova and Pedersen, 1998; Kotelnikova, 2002; Lever, 2012; Kietäväinen and Purkamo, 2015; Lau et al., 2016; Miettinen et al., 2018). Deep groundwaters have been shown to contain these low-molecular-weight key carbon compounds, for example, methanol (∼65 μM) and acetate (∼1.5 mM) in Olkiluoto Finland (Bell et al., 2018), and acetate (1,200–1,900 μM) and formate (480–1,000 μM) in the fracture waters of the Kidd Creek Mine, Canada (Sherwood Lollar et al., 2021). Abiotic synthesis could supply these essential organic carbon compounds to microbial communities (Sherwood Lollar et al., 2021), or they can be produced biotically by microorganisms. Acetate is an important compound in microbial metabolism. It can be formed by autotrophic acetogens, by SRB in incomplete oxidation of organic carbon compounds, or released in organic carbon degradation (Lovley and Chapelle, 1995; Lever, 2012; Pedersen, 2012); it can be further assimilated as a carbon source into biomass in the deep subsurface (Nuppunen-Puputti et al., 2018), or it can be consumed by acetoclastic methanogens (Whitman et al., 2006; Escudero et al., 2018a). Methane is abundant in the deep bedrock groundwaters in Outokumpu, and it is the dominant dissolved gas at the depth of 500 m with a concentration of 22 mmol/L groundwater (Kietäväinen et al., 2013). Based on isotopic, microbial, and metagenomic studies, it has been suggested that the methane found in the shallower parts of the deep drill hole originates dominantly from biological methanogenesis (Kietäväinen, 2017; Kietäväinen et al., 2017). Furthermore, methanol could be formed in the deep subsurface by methanotrophic microorganisms through methane oxidation (Hanson and Hanson, 1996; Kotelnikova, 2002). Formed methanol can then be oxidized to formaldehyde and further into formic acid (Hanson and Hanson, 1996). The Outokumpu deep subsurface planktic microorganisms have been shown to transfer carbon from acetate or carbonate into their biomass and nucleic acids (Bomberg et al., 2017; Nuppunen-Puputti et al., 2018) and respond fast to introduced methanol and methane turning from hibernation into an active state (Rajala et al., 2015; Rajala and Bomberg, 2017). Whether and how these essential carbon compounds activate and sustain microbial rock surface communities in a similar manner remains unresolved. Metagenome assembled genomes (MAGs) from mica schist containing microcosms with Outokumpu deep groundwater indicated that besides heterotrophy, sulfur cycling, such as sulfur oxidation and sulfate reduction, as well as saprotrophic necromass scavenging lifestyles could be common in the sessile epilithic bacterial communities (Nuppunen-Puputti et al., 2022). Moreover, the deep continental subsurface endolithic and epilithic microbial community metagenomes and MAGs contained genes for the oxidation of methane and methanol, as well as acetate metabolism (Nuppunen-Puputti et al., 2022; Sahu et al., 2022). In addition to bacteria, fungi have also been shown to occupy both mica schist surfaces and the water phase *in situ* and in microcosm studies from the Outokumpu deep subsurface (Nuppunen-Puputti et al., 2021, 2022). Epilithic fungi occupied up to ∼10% of the surface area of *in situ* enriched rock coupons in the DeMMO, USA (Casar et al., 2021), and they were also part of the endolithic microbial communities in Äspö, Sweden (Ekendahl et al., 2003; Schäfer et al., 2015; Drake et al., 2017). Analysis of fungal isolates from the oceanic deep subsurface combined with metatranscriptome analysis indicated that deep biosphere fungi likely take part in cycling organic matter (Quemener et al., 2020). However, the role of epilithic fungi in the deep continental biosphere biogeochemical cycles remains unresolved.

In this study, we aimed to examine how a short pulse of simple, low-molecular-weight carbon compounds guides the development of bacterial, archaeal, and fungal communities and their biofilm formation on mica schist surfaces in the microcosms We estimated the abundance of these communities through quantitative polymerase chain reaction (qPCR) analysis and analyzed the indicator species related to the added carbon compounds based on amplicon sequencing of taxonomical marker genes. As genes related to sulfur cycling were observed in the sessile mica schist communities previously (Nuppunen-Puputti et al., 2022), we estimated the abundance of epilithic and planktic sulfate-reducing bacteria (SRB) with dissimilatory sulfite reductase beta subunit gene (*dsr*B) targeting qPCR.

## 2. Materials and methods

### 2.1. Site description

The field site is located in the North Karelia region in Outokumpu, Finland (62.72 N, 29.07 E). The Outokumpu scientific deep drill hole has been drilled for scientific purposes and has a steel-pipe casing for the top 40 m. The deep drill hole reaches a vertical depth of 2,516 m and passes through several open fracture zones enabling fluid flow exchange with the surrounding bedrock. In this study, the fracture zone at the depth of 500 m was sampled for deep fracture fluids during week 42 in October 2010. Prior to the sampling, inflatable packers were installed in order to isolate the fracture zone, which was purged by pumping for 3 weeks to enable the collection of native groundwater, i.e., fracture fluid. Changes in geochemistry and in the structure of the microbial communities were monitored over the time of pumping (Purkamo et al., 2013; Kietäväinen, 2017). The microbial community and their functionality have been described previously (Itävaara et al., 2011; Purkamo et al., 2013, 2014, 2016; Nyyssönen et al., 2014; Rajala et al., 2015; Bomberg et al., 2017; Rajala and Bomberg, 2017; Nuppunen-Puputti et al., 2018, 2021, 2022). The lithology of the deep drill hole has been described earlier indicating mica schist as the dominating rock type at the 500-m depth (Västi, 2011). Hydrogeochemistry of the deep groundwaters has been thoroughly analyzed, and both hydraulic testing and temperature logs have indicated fluid flow within bedrock (Ahonen et al., 2011; Kukkonen et al., 2011; Kietäväinen et al., 2013, 2014; Sharma et al., 2016; Kietäväinen, 2017; Nuppunen-Puputti et al., 2022). The Outokumpu deep subsurface waters have been divided into five divergent water types of which the 500-m fracture zone waters belong to the saline Na-Ca-Cl-dominated water type II with methane (22 mmol/L) and nitrogen (6.1 mmol/L) as the main gases (Kietäväinen et al., 2013). The fracture zone located at the depth of 500 m has a hydraulic conductivity of 3.5 × 10^–7^ m/s (Sharma et al., 2016). The bacterial 16S rRNA gene copy count in the 500-m depth fracture fluid was 1.88 × 10^6^/ml (with a standard error of mean 2.99 × 10^5^), and the archaeal 16S rRNA gene copy count was 8.6 × 10^1^/ml (with a standard error of mean ± 1.23 × 10^0^) in previous studies (Purkamo et al., 2016). Furthermore, the *dsr*B gene copy number of the 500-m depth fracture fluid was 7.4 × 10^3^/ml (Purkamo et al., 2013).

### 2.2. Experimental setup and sampling

Microcosms with crushed mica schist were prepared as previously described (Nuppunen-Puputti et al., 2022). Drill core mica schist, derived from a depth of 506.1 m, Outokumpu, was crushed with a ball mill and sieved (grain size < 5 mm) (Västi, 2011; Nuppunen-Puputti et al., 2022). A total of 3 g of mica schist was weighted into 120-ml headspace bottles and autoclaved (121°C, 15 min). Microcosms were prepared in triplicates in an anaerobic field hood as described previously (Nuppunen-Puputti et al., 2022). Briefly, anoxic groundwater was pumped through a polyamide tube (diameter 10/12 mm) from the isolated fracture zone with a membrane pump directly into an anaerobic field glove box. Deep groundwater was first collected into sterile (acid-washed, autoclaved) Schott bottles (Schott Duran, Wertheim/Main, Germany), and then, 80 ml of it was further aliquoted into the microcosms containing autoclaved crushed mica schist (Table 1). The microcosms were transported to the laboratory in cold boxes and kept at +8°C until the incubation started a week later, right after substrate additions were done in the laboratory. Triplicate microcosms containing 80 ml of sampled fracture fluid with 40 ml volume for headspace were supplied with 9 ml of methane, and 0.2 ml of methanol or acetate was added to a final concentration of 1 mM. Sulfate in the form of Na_2_SO_4_ was added to the acetate-amended microcosms to a final concentration of 0.75 mM. Three microcosms remained without added substrates. All microcosms contained crushed mica schist. In addition, two microcosms containing distilled sterile water and sterilized mica schist were prepared as abiotic mica schist and processing controls. The experiment included also 1 L duplicate samples representing the original fracture fluid without amendments collected at the field site in the anaerobic chamber on 0.22 μm pore-sized cellulose acetate bottle-top filters (Corning, NY, USA). Sample filters were then cut from the funnels with sterile scalpels, put in sterile plastic tubes, and kept on dry ice until transferred into storage at -80°C at the laboratory.

**TABLE 1:** Sample IDs, sample type, collected sample phase representing either original fracture fluid or the microcosms (mica schist and water), and added substrate.

Sample ID	Sample type	Collected phase	Substrate
FF_A	Fracture fluid	Fracture fluid	No
FF_B	Fracture fluid	Fracture fluid	No
NS_A	Microcosm	Mica schist	No
NS_B	Microcosm	Mica schist	No
NS_C	Microcosm	Mica schist	No
CH4_A	Microcosm	Mica schist	Methane
CH4_B	Microcosm	Mica schist	Methane
CH4_C	Microcosm	Mica schist	Methane
AS_A	Microcosm	Mica schist	Acetate, sulfate
AS_B	Microcosm	Mica schist	Acetate, sulfate
AS_C	Microcosm	Mica schist	Acetate, sulfate
MeOH_A	Microcosm	Mica schist	Methanol
MeOH_B	Microcosm	Mica schist	Methanol
MeOH_C	Microcosm	Mica schist	Methanol
NS_AW	Microcosm	Water	No
NS_BW	Microcosm	Water	No
NS_CW	Microcosm	Water	No
CH4_AW	Microcosm	Water	Methane
CH4_BW	Microcosm	Water	Methane
CH4_CW	Microcosm	Water	Methane
AS_AW	Microcosm	Water	Acetate, sulfate
AS_BW	Microcosm	Water	Acetate, sulfate
AS_CW	Microcosm	Water	Acetate, sulfate
MeOH_AW	Microcosm	Water	Methanol
MeOH_BW	Microcosm	Water	Methanol
MeOH_CW	Microcosm	Water	Methanol
MSCNeg1	Abiotic microcosm	Sterile mica schist, sterile water	No
MSCNeg2	Abiotic microcosm	Sterile mica schist, sterile water	No

One liter original situation representing fracture fluid sample replicates were collected and filtered on site at the field laboratory (FF), whereas incubated 80-ml microcosm rock or water phase samples were collected in the laboratory at the end of the experiment after 3.5 years enrichment. NS-coded samples represent treatments without added substrates, CH4, methane, AS, acetate and sulfate, MeOH, methanol. Both planktic and mica schist phases were combined into one filter for the negative mica schist controls containing sterile milli-Q water as well as sterile mica schist (MSCNeg).

### 2.3. Incubation of the microcosms and DNA extraction

Microcosms were incubated statically in the dark for approximately 40 months (∼3.5 years) at +11°C, which is comparable to the *in situ* temperature at the depth of 500 m in Outokumpu (Ahonen et al., 2011; Kukkonen et al., 2011). At the end of the incubation, pH in the microcosm water phase was measured with a basic pH probe (Denver Instruments, Bohemia, NY, USA). The 80 ml planktic water phase microbial communities were collected on Corning cellulose acetate (CA) filters (Corning, NY, USA). This filter was cut into smaller pieces (3 mm × 3 mm) on a sterile Petri dish with a sterile scalpel, transferred into a DNA extraction tube, and frozen at -20°C until further handling. The remaining mica schist crush was transferred into a 50-ml Corning centrifugation tube (Corning Inc., New York, NY, USA) and first double washed gently with sterile 0.9% NaCl solution that was carefully removed by pipetting and discarded. The rock surface biofilm was then detached from the mica schist with 5 ml of 1 × PBS amended with 5 μl of Tween 20 (Bio-Rad, Hercules, CA, USA). The crushed mica schist samples were shaken for 20 min at 150 rpm and then sonicated for 3 min in a water bath at ambient temperature. The PBS solution was pipetted into a new sterile plastic 15-ml centrifuge tube (Corning Inc., New York, NY, USA) and centrifugated (3,200 *g* for 10–30 min). The supernatant was carefully removed, and finally, the pellet was suspended with 100 μl of SL1-buffer (Macherey-Nagel, Düren, Germany). The suspended sample was transferred into a DNA extraction tube and frozen at -20°C until further handling. DNA extraction was performed with the NucleoSpin Soil DNA kit (Macherey-Nagel, Düren, Germany) by following the protocols provided by the manufacturer. The acquired DNA was quantified with a NanoDrop 1000 spectrophotometer (NanoDrop Technologies Inc., Wilmington, DE, USA).

### 2.4. Preparation of amplification libraries for iSeq100 sequencing

Samples for amplicon library sequencing with the Illumina iSeq100 were prepared as previously described (Nuppunen-Puputti et al., 2022). Bacterial amplicon sequence libraries were prepared with Bact_341F/Bact_805R primers (Herlemann et al., 2011), fungal amplicon sequence libraries were prepared with ITS1/ITS2 primers (White et al., 1990; Gardes and Bruns, 1993), and archaeal amplicon sequence libraries were prepared with S-D-Arch-0349-a-S-17/S-D-Arch-0787-a-A-20 primers (Klindworth et al., 2013). The master mix for duplicate reactions contained 1 × MyTaq Red Mix (Bioline, London, UK), 1 μl of each 20 μM primer, 2 μl of sample DNA, and nucleic acid-free water filled to the reaction volume of 25 μl. For all libraries, the same amplification program was followed: initiation for 3 min at 95°C; 40 repetitive 15 s amplification cycles at 95, 57, and 72°C each; last elongation 30 s at 72°C; and final cooling. Pooled amplicons were purified with the NucleoMag NGS Clean-up and Size Select magnetic beads and NucleoMag SEP magnet (Macherey-Nagel, Düren, Germany) in U-shaped 96-well plates (4titude, Surrey, UK). First, the magnetic-bound samples were washed two times with 80% ethanol; then, ethanol was removed carefully by pipetting and 10 min desiccation; and finally, the purified samples were eluted from the beads with 10 mM Tris, pH 8.5 (bioPLUS Buffers and Reagents, Dublin, Ohio, USA). The purified amplicons were indexed with the Nextera XT v2 kit (Illumina, Inc., San Diego, CA, USA), index C for bacteria, index D for fungi, and index B for archaea. The indexing PCR was performed with an Eppendorf Mastercycler (Eppendorf, Hamburg, Germany) in 25 μl reactions. In detail, the PCR mix contained 1 × MyTaq Red HS Master Mix (Bioline, London, UK), 2.5 μl of indices, 1 μl of DNA, and 5 μl of nuclease-free water (Sigma-Aldrich, St. Louis, MO, USA). Indexing started with 30 s at 95°C, followed by eight repetitive cycles for 30 s at each temperature, i.e., 95°, 57°, and 72°C, final elongation for 5 min at 72°C, and cooling. The indexed amplicons were purified again with the size selective beads and diluted with Tris to 1: 100,000 dilutions. These dilutions were used for qPCR performed with the JetSeq Hi-ROX library quantification kit (Bioline, London, UK) in 10 μl reaction volumes in order to determine the sample concentrations for pooling. The qPCR program commenced with 2 min at 95°C and continued with 35 repeated cycles of 5 s at 95°C and 60 s at 60°C, melting curve analysis, and cooling. The correction for amplicon size vs. standard size was done with 550 bp amplicon size for the bacteria and archaea, and 500 bp amplicon size for the fungi. The samples were equimolarly pooled into suitable mixes. The mixes were run in 1% agarose gel electrophoresis with 1 × SB buffer at 120 V for an hour; right-sized products were excised from the gel and purified with the XS gel purification kit (Macherey-Nagel, Düren, Germany) with a final elution volume of 12 μl. The purified mixes were diluted to 1:1,000 and 1:10,000 on 96-well plates (4titude, Surrey, UK) and used in the JetSeq Hi-ROX qPCR analysis to determine the concentration of the final libraries. The final library mix was adjusted mix-wise (i.e., index plates B, C, and D) according to the number of samples included in each library and their concentrations. We targeted a 5% PhiX Control V3 concentration (Illumina, Inc., San Diego, CA, USA) in the final library mix in order to add more diversity to the samples. Finally, 20 μl of the 110 pM final library mix was loaded into an iSeq100 i1 v2 reagent cartridge and iSeq100 v1 flow cell (Illumina, Inc., San Diego, CA, USA).

### 2.5. Quantitative polymerase chain reaction (qPCR)

The sizes of bacterial, sulfate-reducing bacterial (SRB), archaeal, and fungal communities on mica schist and in water samples after 3.5 years of incubation were evaluated with qPCR analyses performed with the LightCycler 480 (Roche Diagnostics, Basel, Switzerland). All analyses were performed in triplicate 10 μl reactions containing 1 μl of DNA template in a white-walled 96-well qPCR plate (4titude, surrey, UK). The bacterial 16S rRNA gene was targeted with Bact_341F/Bact_805R primers (Herlemann et al., 2011). Furthermore, sulfate-reducing bacteria (SRB) were targeted by the dissimilatory sulfite reductase beta subunit gene (*dsr*B) with the DSRp2060F and DSR4r primers (Wagner et al., 1998; Geets et al., 2006). The SensiFAST SYBR No-ROX 2 × Master Mix (Bioline, London, UK) was used for the bacterial 16S rRNA gene qPCR, and for the SRB, the KAPA Sybr Fast qPCR Master Mix (KAPA Biosystems, Wilmington, MA, USA) was used. The reaction mixes for both qPCR assays contained 0.5 μM of each primer and molecular grade water to a reaction volume of 10 μl. The qPCR program began with an initial denaturation at 95°C for 15 min, then 40 or 45 repeated amplification cycles with denaturation at 95°C for 10 s, annealing at 57°C for 35 s, and elongation at 72°C for 30 s were run for bacteria and SRB, respectively, and the program was finalized with a final elongation at 72°C for 3 min, melting curves analysis, and cooling to 40°C. The fungal 5.8S rRNA gene was targeted with the 5.8F1/5.8R1 primers and the FAM-labeled probe 5.8P (Haugland and Vesper, 2002). Archaeal 16S rRNA gene was targeted with A344F/A744R primers (Barns et al., 1994; Bano et al., 2004) and archaea-specific FAM-labeled probe A516F (Takai and Horikoshi, 2000) as previously described (Bomberg and Miettinen, 2020). The qPCR was performed using the SensiFAST SYBR No-ROX 2 × Master Mix (Bioline, London, UK) in reactions containing 0.5 μM of each primer, 0.2 μM probe, and molecular grade water to 10 μl. The qPCR program for the archaea began with an initial enzyme activation step at 95°C for 3 min and then 40 repeated amplification cycles of 95°C for 10 s, 57°C for 35 s, and 72°C for 1 s. The gene copy numbers were calculated from a 10-fold dilution series of a plasmid standard, which contained *Escherichia coli* 16S rRNA gene for bacteria, the *dsr*B gene of *Desulfobulbus propionicus* for the SRB, the 5.8S rRNA gene of *Aspergillus versicolor* for fungi, and the 16S rRNA gene of *Halobacterium salinarum* for archaea as described in the studies by Rajala et al. (2016) and Bomberg and Miettinen (2020).

### 2.6. Amplicon sequence analysis and microbial community statistics

The sequence data were analyzed in RStudio (v. 1.1.456) with R (v. 4.0.4) (RStudio Team, 2015). Amplicon sequence libraries were analyzed with the DADA2 package v. 1.19.2 (Callahan et al., 2016). The primers were removed prior to analysis with cutadapt. The following parameters were used for trimming the bacterial and fungal sequence reads: minlength = 180; maxN = 0; truncQ = 2; trimLeft = 10; trimRight = 35; for bacteria, maxEE = 5; and for fungi, maxEE = 2. For the archaeal libraries, the trimming parameters of minlength = 150, maxN = 0, truncQ = 2, trimLeft = 10, trimRight = 40, and maxEE = 2 were used. The chimeras were analyzed and removed with the command removeBimeraDenovo. Taxonomy for bacterial and archaeal communities was assigned with Silva v. 138 (Quast et al., 2013), whereas taxonomy for fungal ITS sequences was assigned with UNITE v.8 (Kõljalg et al., 2013; Nilsson et al., 2019). The sampling and processing of the samples were performed carefully with aseptic microbiological principles and techniques; yet, typically to low biomass samples, contaminant assumed microbial genera were recognized from the amplicon data (e.g., *Rhizobium* and *Streptococcus*) during processing. Data cleaning routines suggested by Salter et al. (2014), Sheik et al. (2018), and Fullerton et al. (2021) were applied, with exception of keeping the deep groundwater genera that have been shown to represent the intrinsic microbial population in Outokumpu deep groundwaters in numerous research (e.g., Purkamo et al., 2016; Bomberg et al., 2017). The list of removed typical contaminants potentially arising from the molecular biological kits, humans, or environment is included in the supplements (Supplementary Table 1). In addition, data were compared against amplification controls and ASVs exceeding the average 3% in controls, Chloroplast ASVs and ASVs not matching with the targeted domain were removed from the data. Microbial community structures were visualized with heatmaps using the ampvis2 (Andersen et al., 2018) and ggplot2 packages in R (Wickham, 2016). Alpha diversity measures were estimated with the phyloseq package using raw sequence data (McMurdie and Holmes, 2013). Beta diversity was analyzed with principal coordinates analysis (PCoA) with relative abundance data with the phyloseq package as well as further permutational multivariate analysis of variance (PERMANOVA) testing performed with the vegan package (Anderson, 2001, 2017; McMurdie and Holmes, 2013; Oksanen et al., 2018). The dispersion effects were analyzed with the betadisp and permutest functions from the vegan package (Anderson, 2006; Anderson et al., 2006; Oksanen et al., 2018). The accuracy of the used groupwise sample sizes for multivariate dissimilarity data analysis was checked with a pseudo multivariate dissimilarity-based standard error (multSE) according to modifications in “https://github.com/jslefche/multSE” and with 10,000 repeated samplings (Anderson and Santana-Garcon, 2015; Lefcheck, 2017). Prior to multSE analysis, data were log-transformed with vegan decostand, and then, dissimilarity matrix was built with vegdist command from vegan with method “bray.” Indicator species analysis was performed with the indicspecies package in R (De Cáceres and Legendre, 2009; De Cáceres et al., 2010) with the multipatt-function with func option of either “IndVal.g” for indicator values or “r.g.” for correlation indices, with site group combination parameters duleg = TRUE, min.order = 1, max.order = 4, and with number of permutations control = how(nperm = 99999).

## 3. Results

### 3.1. Microbial community sizes

**FIGURE 1:**
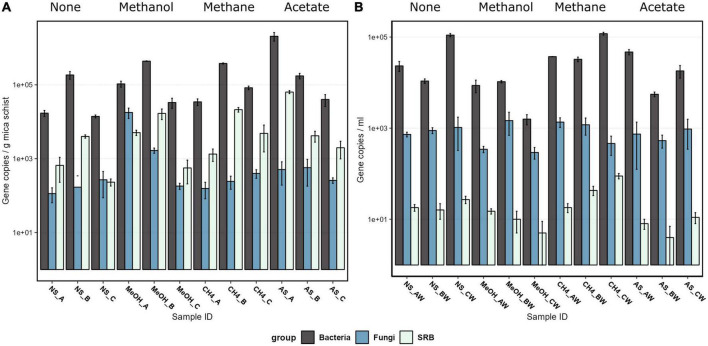
The bacterial 16S rRNA gene, fungal 5.8S rRNA gene, sulfate-reducing bacteria *dsr*B gene, and archaeal 16S rRNA gene copy counts on a logarithmic scale on mica schist surfaces **(A)** and in the microcosm water phase **(B)** as quantified with qPCR. The error bars indicate the standard deviation. Sample name abbreviations are presented in detail in Table 1. FF, fracture fluid; MeOH, methanol; CH4, methane; AS, acetate + sulfate; NS, no substrate; A, B, C, rock surface sample replicates; AW, BW, CW, water phase sample replicates.

Bacteria were the most abundant microbial group both in carbon substrate-supplied microcosms and microcosms without added carbon substrate, according to the qPCR analysis (Figure 1 and Supplementary Table 2). Bacteria occupied both mica schist surfaces and the microcosm water phase. The mica schist-attached bacterial 16S rRNA gene copy counts ranged from 1.4 × 10^4^ to 2.7 × 10^6^ copies/g, whereas the number of *dsr*B gene copy numbers varied between 2.3 × 10^2^ and 6.4 × 10^4^ copies/g mica schist (Figure 1). Furthermore, the ratio of the *dsr*B gene copies to 16S rRNA gene copies was 0.02–0.06 on mica schist. In contrast, planktic bacterial 16S rRNA gene copy numbers from the equivalent microcosms varied between 1.6 × 10^3^ and 1.2 × 10^5^ copies/ml, and planktic *dsr*B gene copy numbers were lower (< 1 × 10^2^ copies/ml). The fungal 5.8S rRNA gene copy counts varied between samples, and the lowest epilithic fungal counts were observed in microcosms without amended carbon and with added methane. The highest epilithic fungal marker gene copy counts were linked to amended methanol or acetate, whereas the highest average planktic fungal marker gene copy counts were linked to the added methane. Supplied carbon compounds supported the growth of the deep subsurface fungi increasing the detected fungal 5.8S rRNA gene copy counts from 1.7 × 10^1^ ± 1.1 × 10^1^/ml in the original fracture fluid water to 8.2 × 10^2^ ± 3.7 × 10^2^/ml in the microcosm water phase and to 1.8 × 10^3^ ± 1.3 × 10^3^/g on mica schist surfaces. Archaeal 16S rRNA genes were not detected in all microcosms by qPCR as marker gene copy counts remained below the reliable detection limit when the most dilute qPCR plasmid standard contained 8.45 × 10^1^/μl archaeal 16S rRNA gene copies.

### 3.2. Bacterial communities

#### 3.2.1. Beta diversity analysis of the bacterial communities

**FIGURE 2:**
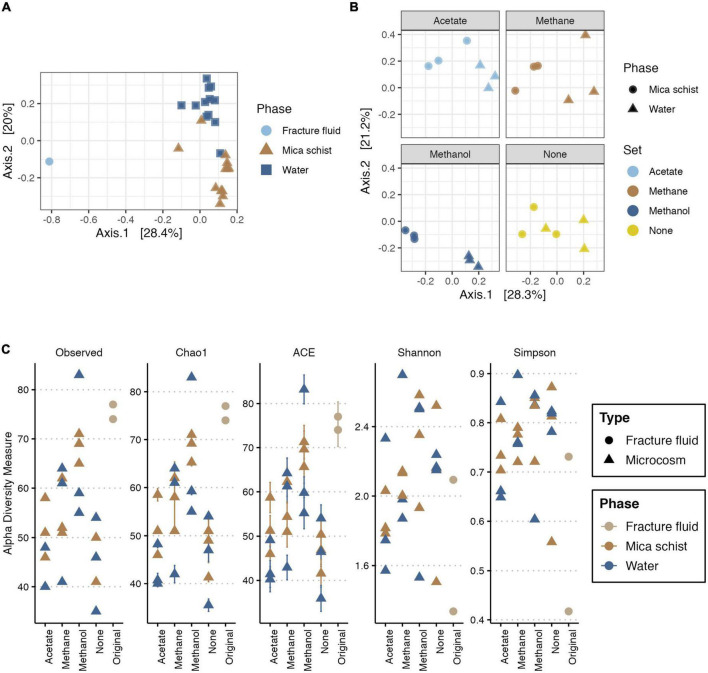
PCoA for bacterial community based on Bray-Curtis’ dissimilarity **(A,B)**, and alpha diversity measures for bacteria **(C)**. For PCoA, axis 1 explains 28.4% **(A)** or 28.3% **(B)**, and axis 2 explains 20% **(A)** or 21.2% **(B)** of the bacterial community variance, respectively. Panel **(A)** includes the original fracture fluid samples, whereas panel **(B)** illustrates dissimilarities in the microcosms. Plot C illustrates the observed number of ASVs, Chao1 and ACE richness estimators, and Shannon and Simpson diversity estimates across sample types. In panel **(A)**, colors and shape indicate the sample phase. In panel **(B)**, colors indicate the amended carbon source, and shape indicates the sample phase. In panel **(C)**, colors indicate sample origin either from fracture fluid or sampled phase in microcosm, and shape indicates the sample type.

Bacterial communities in the original fracture fluid and microcosms differed from each other (Figure 2A, Supplementary Table 3, and Supplementary Figure 1). Observed differences between mica schist attached and planktic phase of the bacterial communities (PERMANOVA:Phase* *R*^2^ = 0.22565, *p* ≤ 0.0001) were more apparent than dissimilarities between microcosms supplied with different simple carbon compounds (PERMANOVA:Set, *R*^2^ = 0.23706, *p* = 0.0056). However, both observed dissimilarities were statistically significant. Replicates clustered more closely for the methanol-amended microcosms indicating higher similarities in the bacterial community structure. Bacterial communities in the microcosms without added substrates also clustered more closely, but the water and rock phase communities of the individual microcosms differed from each other (Figure 2B and Supplementary Figure 1). Furthermore, significant dispersion effects were not observed [permutest for the Phase (*p* > 0.2) and Set (*p* > 0.5)] indicating that detected location effects can be considered relevant (Supplementary Table 4). The accuracy of the sample group sizes used for multivariate dissimilarity analyses for both phase and treatments was adequate as the multSE analysis suggested that both group centroids were calculated with adequate precision (Supplementary Figure 2).

#### 3.2.2. Alpha diversity metrics for the bacterial communities

The bacterial read counts varied between 2,464 and 36,255 across the dataset with 13,651 reads per sample on average (Supplementary Table 5). The number of observed bacterial ASVs ranged from 74 to 77 and from 35 to 83 in the fracture fluid and microcosm samples, respectively (Figure 2C and Supplementary Table 6). Similarly, the predicted richness by estimators Chao1 and abundance-based coverage estimator (ACE) both varied between 74 and 77 in original fracture fluid bacterial communities (with standard errors of ± 0.0 for Chao1 and ± 3.4 to 3.8 for ACE). The methanol-supplied microcosms and the original fracture fluids had the highest ASV richness, whereas the lowest bacterial community richness was observed in microcosms without any substrate additions. In most microcosms, the bacterial community richness decreased during the enrichment compared to the original fracture fluids. There were differences in the bacterial community richness between the mica schist surface and water phase as in some microcosms the planktic community showed higher richness, and in some microcosms, the epilithic community showed higher richness. Shannon and Simpson diversity indices were higher for the microcosm bacterial communities than that for original fracture fluid samples indicating higher bacterial community diversity and evenness after enrichment. In detail, Shannon diversity ranged between 1.3 and 2.1 in the original fracture fluids, and between 1.5 and 2.7 in the microcosm samples, whereas Simpson diversity varied between 0.4 and 0.7 in the original fracture fluids and between 0.6 and 0.9 in the microcosms.

#### 3.2.3. Bacterial community composition

**FIGURE 3:**
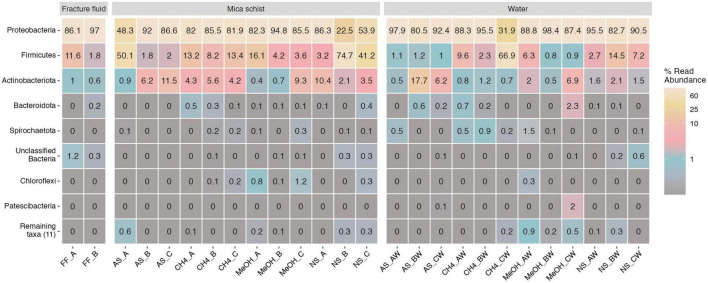
Top 8 bacterial phyla in fracture fluids and different microcosms mica schist or water phase. The remaining taxa show 11 lower abundance phyla and their abundances across treatments and phases. Color scale indicates relative abundance as percentages. Sample name abbreviations are presented in detail in Table 1. A, B, and C in the sample names represent replicate microcosms; W, water phase of the microcosms.

Actinobacteriota (0.6–1% of the bacterial communities), Bacteroidota (0–0.2%), Firmicutes (2–11%), and Proteobacteria (86–97%) phyla, as well as bacteria for which phylum level classifications could not be determined (0.3–1%), were detected in the original fracture fluids (Figure 3 and Supplementary Table 7). The main observed bacterial phyla in the microcosms were Actinobacteriota (0.4–17%), Firmicutes (1–75%), and Proteobacteria (23–97%) (Figure 3 and Supplementary Table 7). Other detected bacterial phyla across microcosms were Bacteroidota, Chloroflexi, Spirochaetota, and unclassified bacteria. All other observed phyla were detected mainly in single microcosm samples (Supplementary Table 7).

**FIGURE 4:**
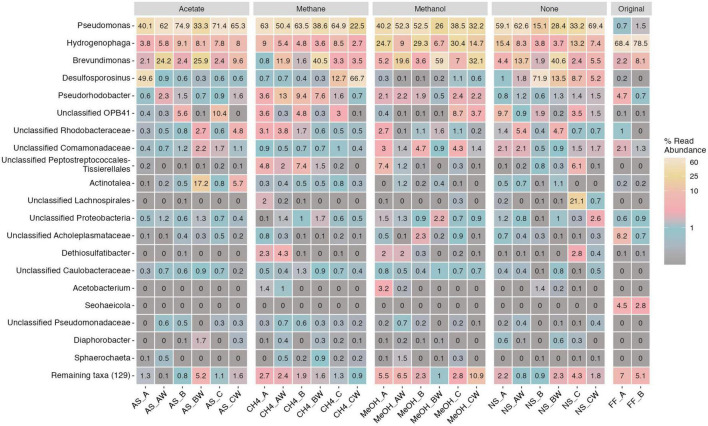
Top 20 bacterial genera across different treatments and samples. Legend color indicates relative abundance (as %). The remaining taxa row shows the remaining 129 lower abundance taxa with their merged relative abundances. Sample name abbreviations are presented in detail in Table 1. FF, fracture fluid; MeOH, methanol; CH4, methane; AS, acetate + sulfate; NS, no substrate; A, B, C, rock surface sample replicates; AW, BW, CW, water phase sample replicates.

The main bacterial genus detected in the original fracture fluid samples was *Hydrogenophaga* (68–79% of the bacterial communities). Other major bacterial genera were observed in the fracture fluid samples affiliated with *Brevundimonas* (2–8%), *Pseudorhodobacter* (1–5%), *Seohaeicola* (3–5%), unclassified Acholeplasmataceae (1–8%), unclassified Comamonadaceae (1–2%), and *Pseudomonas* (1–2%). The main bacterial genera observed across the microcosms were *Brevundimonas* (1–41%), *Desulfosporosinus* (0.1–72%), *Hydrogenophaga* (4–30%), *Pseudomonas* (15–75%), and *Pseudorhodobacter* (0.5–13%) (Figure 4). In addition, the relative abundance of some genera, such as unclassified OPB41 (0–10%) and *Dethiosulfatibacter* (0–4%), was higher in some microcosms. Many genera remained unclassified indicating that a major part of the bacterial community represents previously unidentified potentially new genera. Comparing the relative abundance of the detected genera in original fracture fluid samples to that of the microcosms, it was shown that the enrichment favored *Pseudomonas, Brevundimonas, Desulfosporosinus*, and unclassified OPB41. In addition, some genera were not detected in fracture fluid samples prior to the enrichment, such as *Sphaerochaeta*, likely representing more rare, low-abundance taxa in the deep groundwaters. The eight *Hydrogenophaga* sp. affiliating ASV sequences were compared against the NCBI nr database using BLAST, and two of these ASVs (ASV12, ASV20) matched closest to *Serpentinomonas raichei* (100% similarity) (Supplementary Table 8). We will henceforth discuss this group as *Hydrogenophaga/Serpentinomonas* as these ASVs likely represent both genera.

#### 3.2.4. Indicator species analysis for bacteria

Indicators for different sample types and treatments were investigated with both genus and ASV level indicator species analysis in order to determine in detail which genera/ASVs were linked to the observed differences between differently supplied microcosms. In addition, indicator species for sessile mica schist surface or planktic phase were examined separately. *Pseudomonas* was a significant indicator species for the acetate-amended microcosms (*p* ≤ 0.016), and *Proteiniphilum* was a significant indicator species for the methane-amended microcosms (*p* = 0.0004) (Supplementary Tables 9, 10). In addition, *Pseudorhodobacter* was a significant indicator when considering correlation indices for the methane-amended microcosms (*p* = 0.03) (Supplementary Table 10). Original fracture fluids hosted a variety of significant indicator species, such as *Phenylobacterium*, *Hydrogenophaga/Serpentinomonas*, and unclassified Desulfuromonadaceae (*p* = 0.003 for all). *Desulfosporosinus* (*p* = 0.008), unclassified Caulobacteraceae (*p* = 0.003), and *Pseudomonas* (*p* = 0.002) were individual indicator species for all microcosms. *Pseudomonas* ASV2 (*p* < 0.001) was an indicator in acetate-amended microcosms at ASV level. *Hydrogenophaga* ASV6 (*p* = 0.003) and *Hydrogenophaga/Serpentinomonas* ASV12 (*p* = 0.004) were important individual indicator ASVs for the methanol-amended communities according to indicator values, whereas statistically significant indicator ASVs in methane-supplied microcosms were unclassified Microbacteriaceae ASV110 (*p* < 0.001), *Pseudorhodobacter* (*p* < 0.006), and unclassified Proteobacteria ASV150 (*p* < 0.006). Sessile unclassified Comamonadaceae (*p* = 0.016), Acholeplasmataceae affiliating EUB-322 (*p* = 0.018), *Brevundimonas* (*p* = 0.036), unclassified Thermotaleaceae (*p* = 0.018), and *Hydrogenophaga/Serpentinomonas* (*p* = 0.018) were significant individual indicators for methanol-supplied mica schist. *Proteiniphilum* was significant for the planktic phase of the methane-supplied microcosms (*p* = 0.01).

### 3.3. Fungal communities

#### 3.3.1. Beta diversity analysis of the fungal communities

**FIGURE 5:**
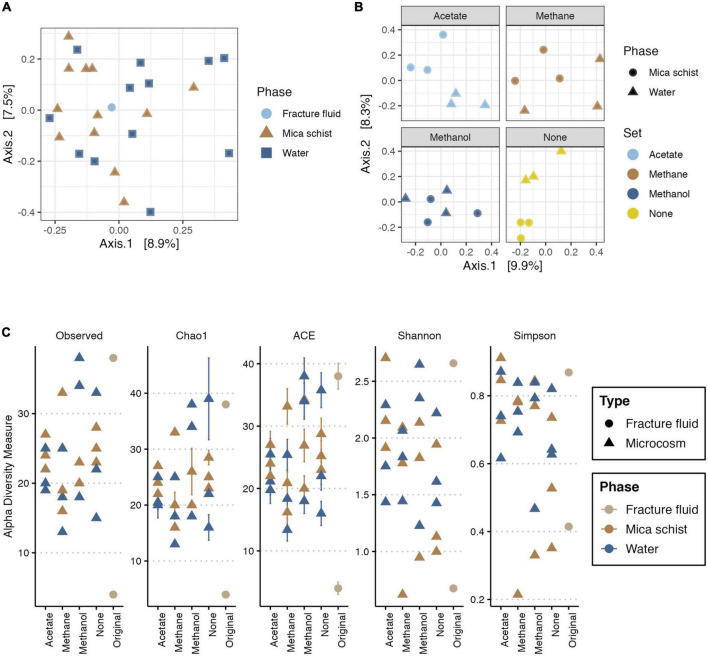
PCoA for fungal community based on Bray-Curtis’ dissimilarity **(A,B)**, and alpha diversity measures for fungi **(C)**. In PCoA, *X*-axis explains 8.9% **(A)** or 9.9% **(B)**, and *Y*-axis explains 7.5% **(A)** or 8.3% **(B)** of variance in the fungal community composition, respectively. Panel **(A)** includes the original fracture fluid samples, whereas panel **(B)** illustrates dissimilarities in the microcosms. In panel **(A)**, colors and shape indicate the sample phase. In panel **(B)**, colors indicate the amended carbon source, and shape indicates the sample phase. Plot C illustrates the observed number of ASVs, Chao1 and ACE richness estimators, and Shannon and Simpson diversity estimates in different sample types and phases. Colors indicate sample origin either from fracture fluid or sampled phase in microcosm.

The fracture fluid and microcosm fungal communities clustered closely together indicating a more similar community composition as Axis 1 explained only a small part of the observed dissimilarities (Figure 5). Yet, minor dissimilarities were observed (PERMANOVA:Type, *R*^2^ = 0.05545, *p* = 0.006) (Supplementary Table 3). The mica schist attached and microcosms planktic fungal communities showed some minor dissimilarities in the community structure (PERMANOVA:Phase, *R*^2^ = 0.09581, *p* = 0.028), which were more clear for the microcosms without added carbon compounds and for the acetate and methane supplied microcosms (Figure 5B and Supplementary Figure 4). In addition, supplied carbon compounds induced some significant dissimilarities in the microcosm fungal community composition (PERMANOVA:Set, *R*^2^ = 0.17854, *p* = 0.05) as different carbon source additions explained ∼18% of the observed variance between fungal communities in different microcosms. The fungal communities in methanol-supplied microcosms clustered more closely indicating higher similarity, yet fungal community structures in different phases of individual microcosms separated (Figure 5 and Supplementary Figure 4). However, multivariate homogeneity of dispersions was statistically relevant for the fungal communities regarding different sample types (permutest, Type, *p* = 0.001) and sampled phases (permutest, Phase, *p* = 0.002), whereas no significant betadispersion effects were observed regarding different treatments (permutest, Set, *p* > 0.1) (Supplementary Table 4). In addition, the estimated sample group sizes for multivariate analysis of fungal communities were adequate (Supplementary Figure 3).

#### 3.3.2. Alpha diversity metrics for the fungal communities

The fungal read counts varied between 301 and 7,695 across the dataset with an average of 2,210 reads per sample (Supplementary Table 5). The number of observed ASVs was lowest in the methane-amended microcosms (13–33), and it was highest in the methanol-amended microcosms (18–34) (Figure 5C and Supplementary Table 11). The richness estimators Chao1 and ACE were in line with the observed ASV counts and predicted highly similar ASV richness across sample types. Shannon and Simpson diversity indices estimated the highest diversity and evenness of fungal communities for the acetate-amended microcosms as Shannon’s (H’) ranging from 1.4 to 2.7 and Simpson index varying between 0.7 and 0.9 (Figure 5C and Supplementary Table 11). The fungal community alpha diversity measures for the original fracture fluid samples FF_A and FF_B differed from each other (Figure 5C) as sample FF_B had high diversity and richness, whereas FF_A represented the lowest.

#### 3.3.3. Fungal community composition and indicator species analysis

**FIGURE 6:**
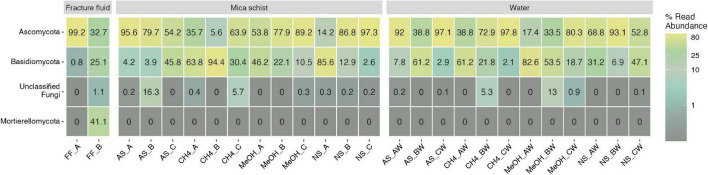
Fungal phyla in fracture fluids and different microcosms mica schist or water phase. The remaining taxa show merged lower abundance phyla and their abundances across treatments and phases. Color scale indicates relative abundance as percentages. Sample name abbreviations are presented in detail in Table 1. A, B, and C in the sample names represent replicate microcosms; W, water phase of the microcosms.

**FIGURE 7:**
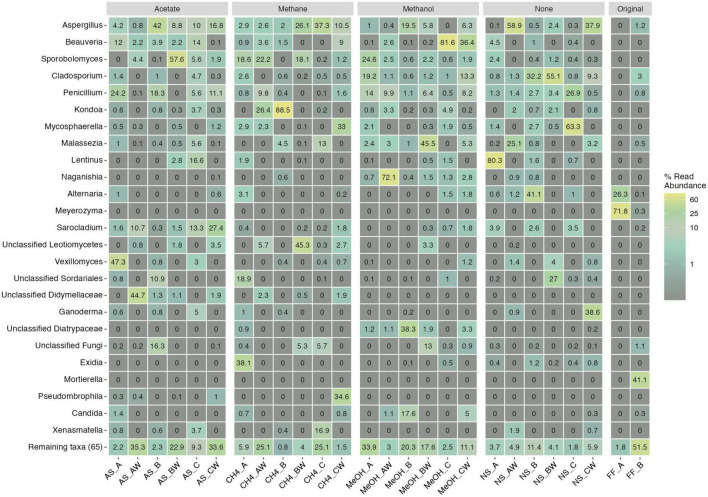
Top 25 fungal genera across different treatments and samples. Legend color indicates relative abundance (as %). The remaining taxa row shows the remaining 65 lower abundance taxa with their merged relative abundances. Sample name abbreviations are presented in detail in Table 1. FF, fracture fluid; MeOH, methanol; CH4, methane; AS, acetate + sulfate; NS, no substrate; A, B, C, rock surface sample replicates; AW, BW, CW, water phase sample replicates.

The main observed fungal phyla across samples were Ascomycota, Basidiomycota, and unclassified fungi for which phylum level classification could not be assigned (Figure 6 and Supplementary Table 7B). In addition, Mortierellomycota was observed in one original fracture fluid sample (Figure 6). The fungal community composition detected in both replicate samples of the original fracture fluids hosted the genera *Alternaria, Meyerozyma*, and *Fusarium* (Figure 7). The fungal community composition in fracture fluids showed great variability as the other sample contained a larger number of different genera. In the microcosms, *Aspergillus, Beauveria, Cladosporium, Penicillium*, and *Sporobolomyces* were common and abundant. *Meyerozyma* and *Fusarium* were statistically significant indicators for fracture fluid samples (*p* = 0.003) (Supplementary Tables 12, 13). *Sarocladium* was a significant indicator fungus in the acetate-amended microcosms (*p* = 0.009). *Aspergillus* ASV17 (*p* = 0.02), *Naganishia* ASV42 (*p* = 0.002), *Malassezia* ASV111 (*p* = 0.01), and *Phaeotremella* ASV113 (*p* = 0.003) represented individual indicator ASVs in methanol-amended microcosms. Unclassified Leotiomycetes was a significant indicator in the planktic phase of methane-amended microcosms (*p* = 0.03), and *Vexillomyces* was a significant indicator in the sessile phase of acetate-amended microcosms (*p* = 0.02).

### 3.4. Archaea

The archaeal 16S rRNA gene copy counts remained below the detection limit in the qPCR analysis. Accordingly, archaeal 16S rRNA gene amplicons were not obtained from most of the microcosms. Nevertheless, archaeal amplicon libraries produced 15 different ASVs. The original 500 m depth fracture fluid community consisted of Nitrosopumilaceae, Methanobacteriaceae, Methanocellaceae, unclassified Woesearchaeales, Methanoperedenaceae, and Methanococcaceae families (Supplementary Table 7C), whereas all the sequences obtained from the microcosms belonged to Methanobacteriaceae. Most of the observed archaeal sequences were detected from microcosms without any added carbon compounds, with the highest number of sequences (16,212) observed in the rock surface sample NS_B. Four rock surface samples with different substrate additions had only very low levels of archaeal sequences (MeOH_C, CH4_A, CH4_B, and AS_C).

### 3.5. Other measurements

The pH in the microcosms at the end of the incubation ranged from 5.5 to 6.8, whereas the original fracture fluid pH at the depth of 500 m have been shown to range from 6.3 to 8.7 during the sampling period in October 2010 (Kietäväinen, 2017; Supplementary Table 14). The highest acquired DNA concentration 5 ng/μl was measured for crushed mica schist from methanol-amended microcosms (Supplementary Table 14).

## 4. Discussion

Lithology is a major driver of the deep subsurface groundwater and biofilm microbiome structure as the main rock type and minerals in turn affect local hydrogeochemistry (Nyyssönen et al., 2014; Casar et al., 2020; Mehrshad et al., 2021; Sahu et al., 2022; Soares et al., 2023). Microcosms mimicking the *in situ* geochemistry offer a possibility to study the development of biofilm communities and their interactions with the available mineral surfaces, and long-term incubations facilitate observations of changing dynamics in the rock–water interphase dwelling microbial communities (Leandro et al., 2018; Sanz et al., 2021; Nuppunen-Puputti et al., 2022). As energy and carbon sources are limiting factors in the deep subsurface as well as in long microcosm incubation simulations, the microbial genera able to use rock surfaces as their nutrient source are more likely to thrive. In this experiment, we investigated the effect of a short pulse of low-molecular-weight key carbon compounds on the epilithic microbial community development for 3.5 years in microcosms containing both mica schist surfaces and fracture fluids.

### 4.1. Sessile and planktic microbiome

There were statistically significant differences between epilithic and water-phase bacterial communities enriched in the microcosms. As no significant betadispersion was detected, these observed differences between sampled phase (rock or water) bacterial communities may be considered relevant and also carried out with adequate sample size for multivariate analyses according to the multSE analysis. In addition, there were indications that different carbon substrate additions also produced statistically significant differences in bacterial community structure. Fungal community composition showed less dissimilarities overall, and dispersion may have affected part of the observed fungal community variance. Furthermore, the enriched microbial communities in the microcosms were derived from the deep groundwater and could differ from the intrinsic biofilm communities. Differentiation of the resident rock surface microbiome and the planktic microbiome has been shown from other deep subsurface locations (Momper et al., 2017; Kraus, 2021). In the serpentinizing Oman subsurface environment, SRB and methanogenic genera diverged between the rock and fluid phase (Kraus, 2021). Moreover, SRB inhabited the fluid phase in the SURF, whereas rock communities had a lower diversity compared to fluid inhabiting microbial communities (Momper et al., 2017).

The fracture fluids hosted a microbial community that as such was not represented in the microcosms. Most of the microcosms also showed some dissimilarities between replicate samples (Figures 2B, 5B). Small differences in the microbial community structure between replicates could develop during enrichment, for example, due to local mineral heterogeneities or uneven distribution of microorganisms in the deep groundwater as sampled volume was 80 ml. The incubation without additional substrates led to the lowest bacterial community richness but did not show any major drop in the relative abundance of opportunistic taxa such as *Pseudomonas.* However, microcosm enrichments indicated that a methanol pulse may enhance the settlement of some microbial taxa, such as *Hydrogenophaga*, EUB33-2, and unclassified Comamonadaceae, on the rock surfaces. The main enriched bacterial genera across microcosms were affiliated with *Pseudomonas, Hydrogenophaga/Serpentinomonas, Brevundimonas, Desulfosporosinus*, and unclassified OPB41. Similar epilithic microbial communities have been cultivated from deep continental subsurface drill cores directly after drilling in the Iberian Pyrite Belt (IPB), Spain (Leandro et al., 2018). These IPB enrichment cultures produced isolates of, e.g., *Pseudomonas, Brevundimonas*, and *Desulfosporosinus*, thus further linking these genera to the deep subsurface epilithic microbiome (Leandro et al., 2018). In our study, the relative abundance of *Brevundimonas* was higher in the water phase but was frequently encountered on mica schist suggesting that this genus inhabits both water and rock surfaces. A *Brevundimonas* MAG (OKU12) studied previously showed that this genus is involved in both the sulfur cycle and organic carbon oxidation (Nuppunen-Puputti et al., 2022), whereas the IPB enrichment isolate *Brevundimonas* sp. T2.26MG-97 genome also contained numerous genes for a fermentative lifestyle (Sanz et al., 2021). *Brevundimonas* has been shown to inhabit deep continental groundwater in Olkiluoto, Finland (Bell et al., 2020), and in basaltic rock cores from deep oceanic crust in the South Pacific Gyre further affirming the presence of this taxon in multiple deep subsurface groundwater and rock microbiomes (Suzuki et al., 2020).

Sessile rock surface microbiome functionality has been linked to processes involved in both the sulfur cycle and heterotrophy in Outokumpu deep bedrock biosphere (Nuppunen-Puputti et al., 2022). The copy numbers of epilithic community *dsr*B genes were approximately two orders of magnitude lower than those of bacterial 16S rRNA gene copies/g mica schist. However, the proportion of SRB in the bacterial communities may be underestimated, because *dsr*B gene copies are usually present in single copies per genome, whereas bacteria may contain up to 15 copies of 16S rRNA genes per genome (Větrovský and Baldrian, 2013; Müller et al., 2015). In Outokumpu deep subsurface, the ratio of planktic *dsr*B gene copy counts to microscopically enumerated total number of cells in the deep groundwater at the depth of 500 m was previously shown to be 1 *dsr*B: 7.7 planktic cells (Purkamo et al., 2013). Sulfur and thiosulfate cycling bacterial genera, such as *Desulfosporosinus* and *Dethiosulfatibacter*, were observed both on mica schist surfaces and in the water phase of the studied microcosms, and SRB formed a substantial proportion of the epilithic bacterial community. The abundance of *dsr*B gene copies was previously shown to increase over a 2-month incubation period in response to acetate addition in the rock-free microcosms containing groundwater from 967 m from Outokumpu (Purkamo et al., 2017). However, considering that in this study enrichment of SRB was observed especially on the rock surfaces, this could indicate that mica schist could be an important factor affecting the SRB community richness. The Outokumpu mica schist has been shown to contain 0.42% carbon and 0.057% sulfur, whereas the reported total sulfur amount in the Outokumpu deep groundwater at 500 m was 3.36 mg/L, and the concentration of sulfate has been below the detection limit (< 50 mg/L) (Västi, 2011; Kietäväinen, 2017; Nuppunen-Puputti et al., 2022). Epilithic *Desulfosporosinus* MAGs derived from Outokumpu were shown to contain the necessary genes not only for sulfate reduction but also for sulfur oxidation, sulfite oxidation, and sulfite reduction, as well as thiosulfate disproportionation (type 1) (Nuppunen-Puputti et al., 2022). As the sulfate levels in the Outokumpu deep groundwater are low, the ability to use different rock-derived sulfur species may serve as a potential driver of the deep subsurface biological sulfate reduction in the epilithic SRB communities. In the mica schist, sulfur occurs mainly in pyrrhotite [Fe_(1–x)_S] and more rarely in pyrite (FeS_2_) and chalcopyrite (CuFeS_2_) (Västi, 2011). Moreover, sulfides in rocks may be leached by the saline groundwater (Na^+^ and Cl^–^), oxidized in anoxic conditions abiotically with ferric iron Fe (III) or by microorganisms, and released as sulfate or other intermediate sulfur species (Müller and Regenspurg, 2017; Jakus et al., 2021; Bao et al., 2022), which is evident in the recent reviews on sulfide mineral–microbe interactions (Bomberg et al., 2021; Ortiz-Castillo et al., 2021; Spietz et al., 2022). Rock surface biofilms could potentially facilitate interactions in the necessary cycling processes between different groups of microorganisms. High levels of rock-associated sulfate-reducing microorganisms have similarly been observed in the Oman drill cores that hosted endolithic resident SRB genera *Desulfovibrio, Desulfomonas*, and *Desulfomonile* (Kraus, 2021). These SRB were accompanied by several genera of methanogenic archaea, whereas the mica schist in our study supported only low levels of archaea mainly affiliating with the family *Methanobacteriaceae.* Furthermore, archaea remained below the detection limit of the qPCR assay. In accordance, archaea were not found to occupy rock surface biofilms in our previous 6-month *in situ* incubation in the Outokumpu subsurface (Nuppunen-Puputti et al., 2021). In addition to sulfur cycling, previous *Desulfosporosinus* MAGs suggested the potential for C1-cycling as genes for formaldehyde oxidation were identified (Nuppunen-Puputti et al., 2022). Formaldehyde is the main intermediate in methane/methanol oxidation (Hanson and Hanson, 1996), and thus, this suggests that *Desulfosporosinus* could gain some secondary benefit from methanotrophic and/or methylotrophic actions of other microorganisms and therefore become enriched in the microcosms.

*Pseudomonas* was enriched in all microcosms, both on the rock surfaces and in the planktic phase. Previously, enrichment of *Pseudomonas* has been observed in deep groundwater microcosms with nitrite/nitrate and sulfate amendment, but without rock (Bell et al., 2020), and enrichment of planktic Pseudomonadaceae has also been linked to the addition of acetate (Purkamo et al., 2017). *Pseudomonas* and *Hydrogenophaga* were major genera in the deep subsurface rock microbiome in SURF further affirming the ability of these genera to inhabit rock surfaces in the deep biosphere (Momper et al., 2017). Moreover, collective analysis of the deep groundwater microbial communities has identified *Pseudomonas* as a globally relevant core taxon within the deep continental subsurface environments (Soares et al., 2023). The low-abundance taxa affiliated with Chloroflexi and unclassified OPB41 were more frequent in the mica schist surface samples than in the water phase according to the amplicon sequences. Previously collected MAGs of these deep rock surface taxa showed that these groups may be involved in organic carbon cycling and hydrogen oxidation in the epilithic microbiome (Nuppunen-Puputti et al., 2022).

Both filamentous fungi and yeasts were enriched on the mica schist surfaces in the microcosms. Many detected fungal genera inhabited both planktic and rock phases of the microcosms, and the supplied organic carbon increased the fungal 5.8S rRNA gene copy numbers on the mica schist surfaces (methanol, acetate), or in the microcosm water phase (methane). Fungi are heterotrophs and are likely adapted to the deep subsurface by either close syntrophy with bacteria or by saprotrophic actions linked to the use of dead biomass. The rock surfaces may act as a facilitator for these interactions. Fungi are also known for their rock-solubilizing skills that could in turn enhance the actions of attached bacteria (Sterflinger, 2000; Gadd, 2007). There are indications that fungi and sulfate reducers would benefit from each other’s metabolisms in the deep subsurface, and our microcosm study further affirms the co-occurrence of SRB and deep biosphere fungi both on rock surfaces and as planktic communities (Drake et al., 2017; Nuppunen-Puputti et al., 2021). Epilithic *Aspergillus, Aureobasidium, Cadophora, Cladosporium, Dioszegia*, and *Penicillium* detected from Outokumpu represent fungal genera that have also been detected or cultured from the crushed rock material from the deep oceanic crust (Quemener et al., 2020). Furthermore, *Beauveria* isolates from the ocean crust could use bacterial and archaeal peptidoglycans (Quemener et al., 2020). Thus, the enrichment of this taxon could be related to the enrichment of overall microbial biomass in microcosms.

### 4.2. Low-molecular-weight carbon compounds and microbial communities

Our recent study showed that genetic potential for oxidation of organic carbon and fermentation was common across mica schist bacterial MAGs, and genetic potential for the usage of C1-compounds was observed in *Desulfosporosinus, Brevundimonas*, and *Pseudomonas* MAGs (Nuppunen-Puputti et al., 2022). This study further investigated how the pulse of these low-molecular-weight carbon compounds supported epilithic community development in microcosms. The rock surface attached *Hydrogenophaga/Serpentinomonas* had a statistically significant response to methanol. In addition, the relative abundances of *Hydrogenophaga/Serpentinomonas* and unclassified Comamonadaceae on mica schist were the highest in the methanol-amended microcosms. *Hydrogenophaga* and *Serpentinomonas* strains have been shown to grow on acetate and formate (Bird et al., 2021). Correspondingly, one mica schist epilithic *Serpentinomonas* MAG had genes for acetate metabolism (Nuppunen-Puputti et al., 2022). In addition to bacteria, supplied methanol supported the richest fungal communities, and indicator species analysis linked *Naganishia* and *Malassezia* ASVs to the addition of methanol in the microcosms. The ribulose monophosphate (RuMP) or serine pathway is used by various methylotrophic bacteria for methanol oxidation, whereas methanol-utilizing yeasts have the dihydroxyacetone pathway for the use of formaldehyde (Hanson and Hanson, 1996). A variety of methanol-assimilating yeasts have been described from soil environments, where the decaying plant materials release methanol (Morawe et al., 2017). The ability to assimilate methanol has been described for some *Naganishia* yeasts isolated from hypersaline marine environment (Fotedar et al., 2018). In the deep subsurface, methanol is a relevant intermediate and could favor the growth of these small yeasts in the microcosms. In our previous *in situ* experiment, *Naganishia* yeasts were enriched on mica schist surfaces (Nuppunen-Puputti et al., 2021). *Malassezia* has been shown to be activated by CO_2_ amendment in fluid samples from the 180-m depth fracture from Outokumpu (Bomberg et al., 2017). Another potential bacteria benefiting from added methanol would be *Pseudomonas*, which was enriched in all microcosms. Previously, an epilithic mica schist-dwelling *Pseudomonas* MAG was shown to host the *mxa*F gene for methanol oxidation (Nuppunen-Puputti et al., 2022). Moreover, methanol amendment has also been shown to activate dormant deep groundwater microbes, especially *Pseudomonas* and Rhodobacteraceae, from a depth of 500 m in Outokumpu (Rajala and Bomberg, 2017).

In the Outokumpu deep groundwaters, methane is the dominant dissolved gas and thus widely available for microorganisms (Kietäväinen, 2017; Kietäväinen et al., 2017). The planktic microbial community has been shown to rapidly respond to increased availability of methane and methanol together with sulfate, resulting in an increase in the transcription of functional genes representing sulfate reducers (*dsr*B), nitrate reducers (*nar*G), and methanotrophs (*pmo*A) (Rajala et al., 2015). In this study, *Proteiniphilum* and *Pseudorhodobacter* also benefited from the added methane. *Proteiniphilum* was solely detected in the methane-supplied microcosms (both rock and water phase) as a low-abundance genus. Previously, *Proteiniphilum* sp. has been enriched in lignocellulolytic degradation bioreactors with simultaneous enrichment of methanogenic archaea (Wu et al., 2021). However, no methane usage-related genes have been described in *Proteiniphilum* genomes, and it is not considered a methanotroph (Wu et al., 2021). Instead, *Proteiniphilum* has been shown to produce acetate, formate, and multiple volatiles, as well as a variety of extracellular enzymes (Kabaivanova et al., 2022). Similarly, analysis of our previous mica schist-derived *Proteiniphilum* MAG (OKU04) suggested the genetic potential for a wide enzyme pool and the potential for acetogenesis and nitric oxide reduction in the deep subsurface (Nuppunen-Puputti et al., 2022). Therefore, the *Proteiniphilum* genus could represent a potential necromass degrader that in this case could act as a key-compound producer in the microbial community. Another indicator for methane-supplied microcosms was *Pseudorhodobacter.* Previously, *Pseudorhodobacter* was shown to occupy both water and mica schist phases in deep groundwater microcosms without substrate amendments (Nuppunen-Puputti et al., 2022). *Pseudorhodobacter* sp. has been isolated from marine environments, and it has been detected as part of the microbial community in terrestrial subsurface sediments (Li et al., 2016; Ohkubo et al., 2020). Considering that enrichment of this genus was observed also in the shallow hard rock aquifer microbial communities after injection of acetate (Lee et al., 2021), low-molecular-weight carbon compounds appear to benefit this genus in different groundwater environments either directly or through cross-feeding. *Pseudomonas* was abundantly present in all microcosm enrichments. Furthermore, *Pseudomonas* was demonstrated to be a statistically significant indicator for acetate-amended microcosms at both genus and ASV levels. *Pseudomonas* has been linked to the active use of acetate at 2,260 m depth in Outokumpu (Nuppunen-Puputti et al., 2018), and the epilithic *Pseudomonas* MAG OKU15 indicated the potential capability of transforming acetate into acetaldehyde through ethanol fermentation (Nuppunen-Puputti et al., 2022). Acetate-linked metabolism has been shown to hold a central role in another granitic deep terrestrial biosphere location in the KSZ, in the Deccan traps, India (Sahu et al., 2022), where enriched endolithic microbial community metagenomes showed potential for the oxidation of methane and methanol, as well as genes for sulfur cycling (Sahu et al., 2022). The fungal indicator for acetate-amended microcosms was *Sarocladium*. Previously, *Sarocladium* fungi have been shown to occupy both rock surfaces and the water phase after a 6-month *in situ* incubation trial at 500 m and 967 m depth in Outokumpu, where *Sarocladium* also positively correlated with sulfate reducer group SRB2 (Nuppunen-Puputti et al., 2021). *Sarocladium* is part of the deep subsurface groundwater community in Romuvaara, Finland (Purkamo et al., 2018). Furthermore, *Sarocladium* fungi inhabit other extreme environments as they are the primary fungal occupiers of glacier forefield soils after ice sheet melting (Dresch et al., 2019) and occupy plant roots in the Atacama Desert salt lakes (Santiago et al., 2018).

## 5. Conclusion

Sessile and planktic bacterial communities of the Outokumpu deep subsurface-derived mica schist groundwater microcosms differed from each other and from the original 500 m depth fracture fluid communities. Especially, sulfate-reducing bacteria were frequent occupiers of the rock surfaces. Several microbial genera occupied both niches and thus could gain a competitive advantage over solely planktic or epilithic lifestyle in the resource-limiting conditions of the Outokumpu deep subsurface. Significant dissimilarities in the community composition could be observed for the bacterial communities with different supplied carbon sources. *Pseudomonas, Brevundimonas*, and *Hydrogenophaga/Serpentinomonas* dominated the communities along with genera affiliated with *Desulfosporosinus, Sphaerochaeta*, and unclassified OPB41. Fungal rock surface genera were affiliated with *Aspergillus, Cladosporium*, and *Penicillium*. Different carbon amendments were selected for different indicator species suggesting that simple carbon compounds may act as a potential driver influencing the microbial community structure. In sessile rock communities, *Hydrogenophaga/Serpentinomonas* and unclassified Comamonadaceae were individual indicators for supplied methanol. When considering both phases of the microcosms, unclassified *Anaerolineae* and *Naganishia* were significant individual indicators in methanol-amended microcosms, and *Pseudomonas* and *Sarocladium* for added acetate. Although the epilithic and planktic bacterial communities differ in their community structure, it is likely that some sort of metabolic cooperation exists between them and with the fungal communities.

## Data availability statement

The datasets presented in this study can be found in online repositories. The names of the repository/repositories and accession number(s) can be found below: https://www.ebi.ac.uk/ena, PRJEB51996.

## Author contributions

MN-P and MB designed the experiments and prepared the original manuscript. MN-P performed the laboratory analyses, the data visualization, and the formal analysis. RK did the sampling, compiling, and interpretation of geochemical data. IK provided the funding and field site management. MB provided the project leadership. All authors contributed to the manuscript editing and approved the final version of the manuscript.
