# Supplementary material for: Implications of a short carbon pulse on biofilm formation on mica schist in microcosms with deep crystalline bedrock groundwater
Source: Front Microbiol. 2023 Feb 2;14:1054084. doi: 10.3389/fmicb.2023.1054084 (PMC9932282; doi:10.3389/fmicb.2023.1054084)
Supplement: Supplementary file 1 [file Data_Sheet_1.PDF]

Supplementary Material

Supplementary Table 1. The list of scanned putative contaminants for the removal, and retained, intrinsic considered previously described taxa native to the Fennoscandian deep groundwaters (marked with # in the front of the genera). This list is modified from the data cleaning procedures suggested and described in Salter et al., 2014, Sheik et al., 2018 and Fullerton et al., 2021 for low biomass and subsurface samples. Genera marked with * are modified from the originals to match the way taxa is written in the ASV list.

Removing the potential human pathogens and contaminants from kits etc. (Salter et al., 2014, Sheik et al., 2018)

"Afipia","Aquabacterium","Asticcacaulis","Aurantimonas","Beijerinckia","Bosea","Bradyrhizobium", # “Brevundimonas”, # “Caulobacter”,"Craurococcus","Devosia","Hoefleae","Mesorhizobium", "Methylobacterium-Methylorubrum" *,"Methylobacterium", # “Novosphingobium “, "Ochrobactrum", "Paracoccus","Pedomicrobium","Phyllobacterium","Rhizobium","Roseomonas","Sphingobium","Sphingomonas", "Sphingopyxis","Acidovorax","Azoarcus","Azospira", # “Burkhoderia”,"Cupriavidus","Curvibacter", "Delftiae","Duganella", "Herbaspirillum","Janthinobacterium","Kingella","Leptothrix","Limnobacter","Massilia", "Methylophilus","Methyloversatilis","Neisseria","Oxalobacter","Pelomonas","Polaromonas","Ralstonia","Schlegelella", "Sulfuritalea","Undibacterium","Variovorax","Acinetobactera","Enhydrobacter","Enterobacter","Escherichia","Nevskia", "Pasteurella","Pseudoxanthomonas","Psychrobacter","Stenotrophomonas","Xanthomonas", "unclassified Acidobacteria Gp2","Aeromicrobium","Actinomyces","Arthrobacter","Beutenbergia", "Brevibacterium","Corynebacterium","Curtobacterium","Dietzia","Geodermatophilus","Janibacter","Kocuria","Microbacterium", "Micrococcus","Microlunatus","Patulibacter","Propionibacterium","Rhodococcus","Tsukamurella","Chryseobacterium", “Dyadobacter","Flavobacterium","Hydrotalea","Niastella","Olivibacter","Parabacteroides","Pedobacter","Prevotella","Wautersiella", "Deinococcus","Abiotrophia","Bacillus","Brevibacillus","Brochothrix","Facklamia","Lactobacillus","Paenibacillus", "Ruminococcus","Staphylococcus", "Streptococcus ", "Veillonella"

Removing the potential human pathogens and contaminants (Fullerton et al., 2021)

"Acinetobacter", # *"Pseudomonas",*"Abiotrophia","Achromobacter","Actinobacillus","Arcanobacterium", "Arcobacter","Babesia","Bacillus","Bartonella","Bordetella","Borrelia","Brodetella","Brucella", # *"Burkholderia",* # *"Campylobacter",* "Capnocytophaga","Chlamydia", # *"Clostridium",* # *"Comamonas",*"Corynebacterium","Coxiella", "Cronobacter","Deinococcus","Dermatophilus","Ehrlichia","Enterococcus", # *"Erysipelothrix",*"Escherichia", "Escherichia-Shigella" *,"Flavobacterium","Francisella","Gardnerella","Granulicatella","Haemophilus","Hafnia", "Halomonas","Helicobacter","Klebsiella","Kocuria","Lawsonia",# *"Legionella",*"Leptospira","Listeria", "Merkel_cell","Micrococcus","Morganella","Mycobacterium","Mycoplasma","Neisseria","Nocardia", "Pasteurella","Photobacterium","Plesiomonas","Propionibacterium""Proteus","Providencia", # *"Pseudomonas",* "Rhodococcus","Rickettsiae","Roseomonas","Rothia","Salmonella","Serratia", # *"Shewanella",*"Shigella", "Sphaerophorus","Staphylococcus","Stenotrophomonas","Streptococcus","Treponema","Vibrio","Yersinia"

Supplementary Table 2. The mean abundances for bacterial 16S rRNA gene, fungal 5.8S rRNA gene and sulfate reducing bacterial *dsr*B gene copies, and their standard deviation (stdev). The *dsr*B gene copy counts in samples marked with *were extrapolated from the standard curve as the lowest available standard had 37 *dsr*B gene copies and the average Cp for samples marked with * was Cp 32±1.5 (standard deviation).

		Mica schist g^{-1}			Water phase ml^{-1}	
**Sample ID**	**Target**	**mean**	**std**	**Sample ID**	**mean**	**std**
NS_A	Bacteria	17100	3143	NS_AW	23292	5742
NS_B	Bacteria	185000	43313	NS_BW	10842	1210
NS_C	Bacteria	13967	1250	NS_CW	110375	8990
CH4_A	Bacteria	34533	7087	CH4_AW	37250	0
CH4_B	Bacteria	379000	13115	CH4_BW	32542	3734
CH4_C	Bacteria	83200	9430	CH4_CW	118875	8563
MeOH_A	Bacteria	105900	19446	MeOH_AW	8671	2665
MeOH_B	Bacteria	438333	8145	MeOH_BW	10488	628
MeOH_C	Bacteria	33200	10177	MeOH_CW	1580	396
AS_A	Bacteria	2070000	546168	AS_AW	47000	6125
AS_B	Bacteria	174333	29501	AS_BW	5558	648
AS_C	Bacteria	40133	14214	AS_CW	18083	5625
NS_A	SRB	657	428	NS_AW	18*	3
NS_B	SRB	4000	406	NS_BW	16*	6
NS_C	SRB	231	50	NS_CW	27*	5
CH4_A	SRB	1330	492	CH4_AW	18*	5
CH4_B	SRB	21211	3007	CH4_BW	43	10
CH4_C	SRB	4821	3301	CH4_CW	89	12
MeOH_A	SRB	5067	845	MeOH_AW	15*	2
MeOH_B	SRB	16778	5388	MeOH_BW	10*	5
MeOH_C	SRB	560	353	MeOH_CW	5*	4
AS_A	SRB	63667	5897	AS_AW	8*	2
AS_B	SRB	4159	1355	AS_BW	3*	2
AS_C	SRB	1974	988	AS_CW	10*	3
NS_A	Fungi	113	49	NS_AW	730	87
NS_B	Fungi	169	173	NS_BW	890	133
NS_C	Fungi	268	181	NS_CW	1034	709
CH4_A	Fungi	156	74	CH4_AW	1360	325
CH4_B	Fungi	242	94	CH4_BW	1188	485
CH4_C	Fungi	397	104	CH4_CW	460	208
MeOH_A	Fungi	17850	5586	MeOH_AW	344	54
MeOH_B	Fungi	1667	270	MeOH_BW	1468	773
MeOH_C	Fungi	181	34	MeOH_CW	293	84
AS_A	Fungi	506	314	AS_AW	742	617
AS_B	Fungi	570	390	AS_BW	533	169
AS_C	Fungi	258	44	AS_CW	958	613

Supplementary Table 3. PERMANOVA for the bacterial and fungal community analysis with adonis (Bray-Curtis distance matrix, 9999 permutations). The column "adonis" indicates the tested set (Type, Set or Phase). Type was for comparing the dissimilarities in microbial community composition between original fracture fluid and microcosms, Set for dissimilarities in different carbon amendments, and Phase* for testing community dissimilarities between mica schist and water phase. For the bacterial data, the Set* and Phase* (marked with *) excluded the fracture fluid samples from the analysis.

Bacteria

adonis	Total	Df	SumsOfSqs	MeanSqs	F.Model	R^2	Pr(>F)
Type		1	1.4785	1.47847	8.9302	0.27119	0.0039
	Residuals	24	3.9734	0.16556		0.72881	
	Total	25	5.4519			1.00000	
Set		4	2.4030	0.60076	4.318	0.44077	0.0001
	Residuals	21	3.0488	0.14518		0.55923	
	Total	25	5.4519			1.00000	
Phase		2	2.3583	1.17926	8.7682	0.43261	0.0001
	Residuals	23	3.0933	0.13449		0.56739	
	Total	25	5.4519			1.00000	
Set*		3	0.9246	0.30819	2.0715	0.23706	0.0056
	Residuals	20	2.9775	0.14878		0.76294	
	Total	23	3.9001			1.00000	
Phase*		1	0.8801	0.88006	.64109	0.22565	0.0001
	Residuals	22	3.0200	0.13727		0.77435	
	Total	23	3.9001			1.00000	

Fungi

adonis	Total	Df	SumsOfSqs	MeanSqs	F.Model	R^2	Pr(>F)
Type		1	0.616	0.61596	1.4089	0.05545	0.0006
	Residuals	24	10.492	0.43719		0.94455	
	Total	25	11.108			1.00000	
Phase		2	1.0643	0.53217	1.2186	0.09581	0.028
	Residuals	23	10.0441	0.43670		0.90419	
	Total	25	11.1085			1.00000	
Set		4	1.9832	0.49581	1.141	0.17854	0.05
	Residuals	21	9.1252	0.43453		0.82146	
	Total	25	11.1085			1.00000	

Supplementary Table 4. Permutest for testing homogeneity of multivariate dispersions for bacterial and fungal communities (Bray-Curtis' distance matrix, 999 permutations). The column "permutest" indicates the tested set (Type, Set or Phase). Type was for comparing the dissimilarities in microbial community composition between original fracture fluid and microcosms, Set for dissimilarities in different carbon amendments, and Phase for testing community dissimilarities between mica schist and water phase. For the bacterial data, the Set* and Phase* (marked with *) excluded the fracture fluid samples from the analysis.

Bacteria

permutest	Total	Df	Sum Sq	Mean Sq	F	Pr(>F)
Type	Groups	1	0.073786	0.073786	7.0603	0.011
	Residuals	24	0.250821	0.010451		
Set	Groups	4	0.05577	0.013943	0.8823	0.504
	Residuals	21	0.33189	0.015804		
Phase	Groups	2	0.05262	0.026312	1.5583	0.223
	Residuals	23	0.38835	0.016885		
Set*	Groups	3	0.01687	0.0056237	0.3387	0.815
	Residuals	20	0.33207	0.0166034		
Phase*	Groups	1	0.01594	0.015940	0.9029	0.366
	Residuals	22	0.38840	0.017655		

Fungi

permutest	Total	Df	Sum Sq	Mean Sq	F	Pr(>F)
Type	Groups	1	0.038547	0.038547	37.447	0.001
	Residuals	24	0.024704	0.001029		
Set	Groups	4	0.020463	0.0051158	2.1708	0.107
	Residuals	21	0.049490	0.0023566		
Phase	Groups	2	0.031838	0.0159189	11.693	0.002
	Residuals	23	0.031312	0.0013614		

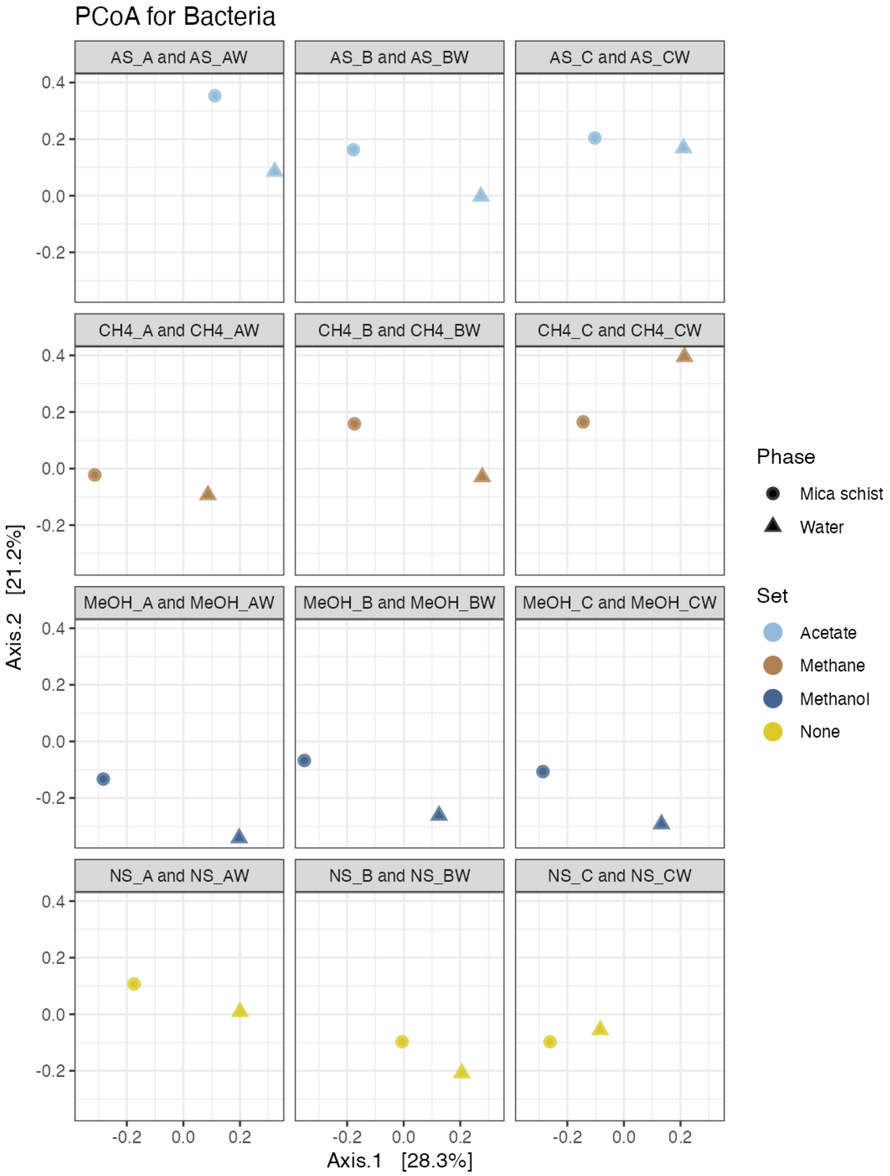

Supplementary Figure 1. The principal coordinate analysis for the bacterial communities. Dissimilarities between different phases in different microcosms without substrate addition or supplied with acetate, methane, or methanol.

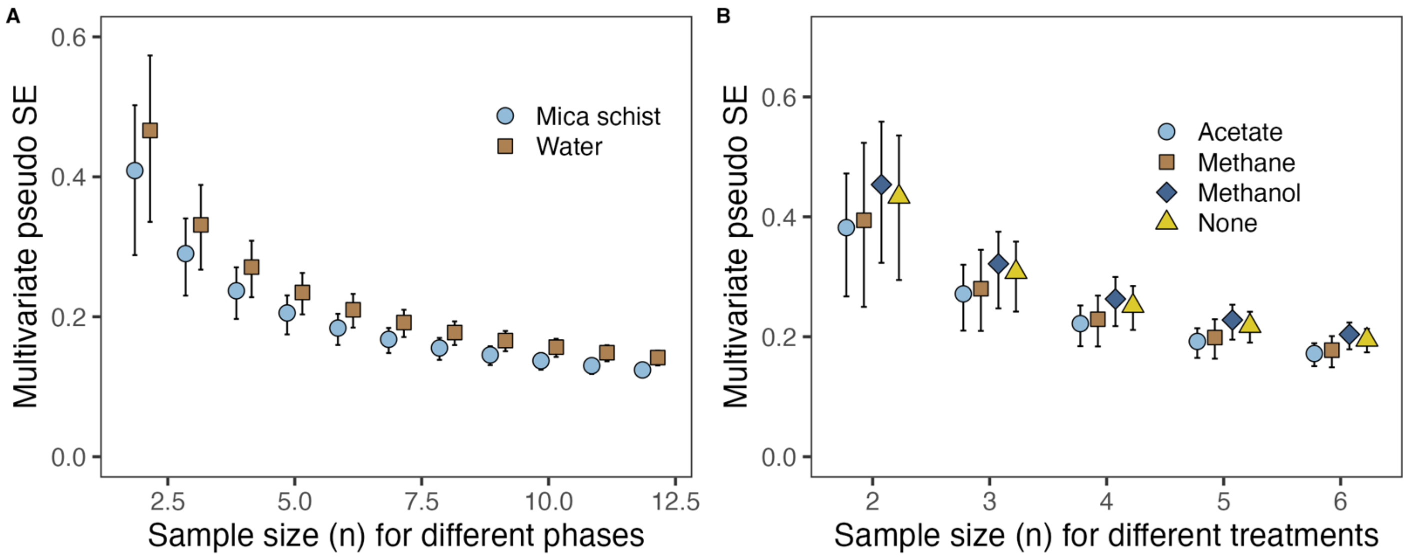

Supplementary Figure 2. Pseudo multivariate dissimilarity based standard error (multSE) analysis for the bacterial communities in the microcosms and estimation of the accuracy of the group sample size for multivariate analysis of A) different phases (Phase*), B) different treatments (Set*).

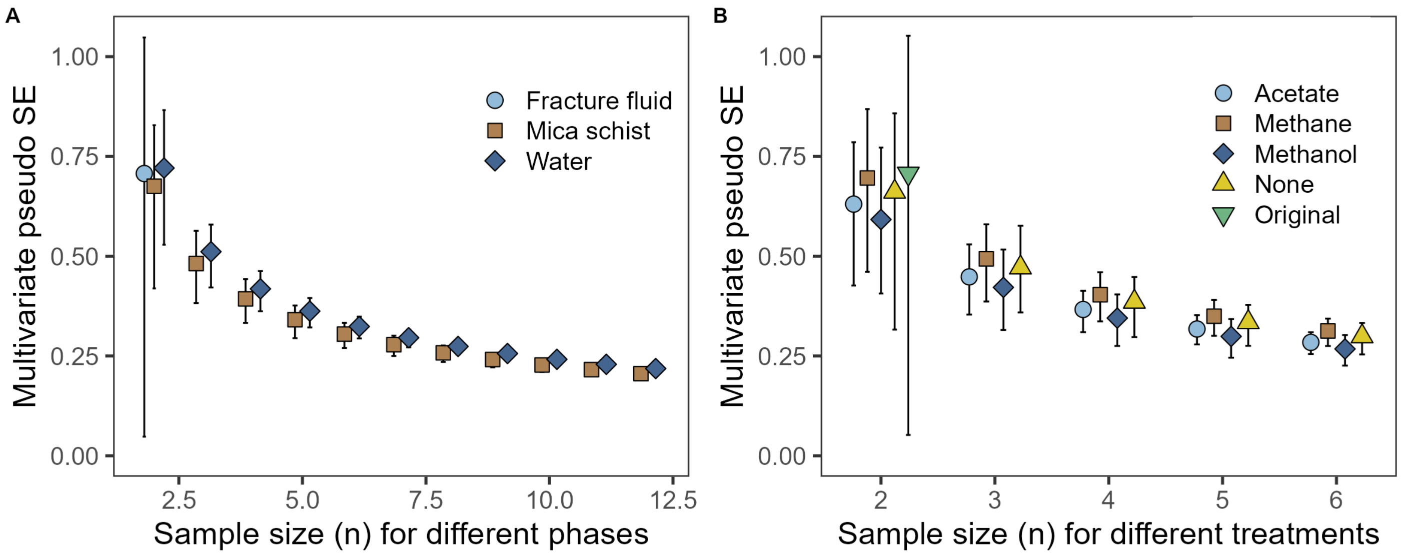

Supplementary Figure 3. Pseudo multivariate dissimilarity based standard error (multSE) analysis for the fungal communities and estimation of the accuracy of the group sample size for multivariate analysis of A) different phases (Phase), B) different treatments (Set) with original fracture fluids included.

Supplementary Table 5. The iSeq100 amplicon library sizes for bacterial 16S rRNA genes and fungal ITS1 regions across samples

	Bacteria	Fungi
FF_A	25483	490
FF_B	30225	6771
NS_A	8776	1931
NS_B	9883	1315
NS_C	7474	3212
MeOH_A	7823	1461
MeOH_B	19643	7695
MeOH_C	13093	990
CH4_A	7046	1642
CH4_B	19909	1421
CH4_C	11584	5528
AS_A	11596	2295
AS_B	14627	2057
AS_C	9954	301
NS_AW	9354	1264
NS_BW	12779	581
NS_CW	2464	3435
MeOH_AW	36255	760
MeOH_BW	12616	1147
MeOH_CW	13376	1371
CH4_AW	13096	387
CH4_BW	13188	3228
CH4_CW	11272	2105
AS_AW	10075	3137
AS_BW	8173	1470
AS_CW	10962	1472
MSC1Neg	10	-
MSC2Neg	175	1458

Supplementary Table 6. Alpha diversity measures for the bacterial communities.

	Observed	Chao1	se.chao1	ACE	se.ACE	Shannon	Simpson
FF_A	74	74	0.00	74	3.8	2.1	0.73
FF_B	77	77	0.00	77	3.4	1.3	0.42
MeOH_A	69	69	0.54	70	4.1	2.6	0.85
MeOH_AW	83	83	0.50	83	3.3	2.5	0.84
MeOH_B	71	71	0.25	71	3.8	1.9	0.72
MeOH_BW	55	55	0.17	55	3.5	1.5	0.60
MeOH_C	65	65	0.74	66	3.7	2.4	0.83
MeOH_CW	59	59	0.74	60	3.6	2.5	0.86
NS_A	46	49	4.62	47	3.2	2.2	0.81
NS_AW	46	47	2.33	46	3.3	2.2	0.82
NS_B	41	41	0.92	42	3.2	1.5	0.56
NS_BW	54	54	0.00	54	3.1	2.1	0.78
NS_C	50	51	2.33	50	3.5	2.5	0.87
NS_CW	35	36	1.29	36	2.9	2.2	0.82
CH4_A	51	51	0.00	51	3.5	2.1	0.72
CH4_AW	64	64	0.50	64	3.4	2.7	0.90
CH4_B	62	62	0.25	62	3.3	2.1	0.79
CH4_BW	61	61	0.17	61	3.7	2.0	0.76
CH4_C	52	58	7.34	54	3.3	2.0	0.78
CH4_CW	41	42	1.81	43	2.8	1.9	0.76
AS_A	46	46	0.00	46	3.2	2.0	0.81
AS_AW	40	41	1.42	41	3.1	1.6	0.65
AS_B	58	58	1.30	59	3.4	1.8	0.70
AS_BW	48	48	0.74	49	3.0	2.3	0.84
AS_CW	40	40	0.16	40	2.9	1.7	0.66
AS_C	51	51	0.12	51	3.4	1.8	0.73

Supplementary Tables 7 and 8. Provided as separate files.

Supplementary Table 9. Indicator values for bacterial communities

Bacteria								
Set	Dataset	Level	Taxa ID	A (specificity)	B (fidelity)	IndVal stat	p-value	significance
Acetate	All	Genus	*Pseudomonas*	0.298	1.0000	0.546	0.0157	*
Fracture fluid	All	Genus	*Sphingorhabdus*	1.0000	1.0000	1.000	0.00328	**
Fracture fluid	All	Genus	*Leucobacter*	1.0000	1.0000	1.000	0.00328	**
Fracture fluid	All	Genus	*Seohaeicola*	1.0000	1.0000	1.000	0.00328	**
Fracture fluid	All	Genus	Unclassified Desulfuromonadaceae	1.0000	1.0000	1.000	0.00328	**
Fracture fluid	All	Genus	*Phenylobacterium*	1.0000	1.0000	1.000	0.00328	**
Fracture fluid	All	Genus	*Silanimonas*	0.9588	1.0000	0.979	0.00328	**
Fracture fluid	All	Genus	Unclassified Burkholderiales	0.8879	1.0000	0.942	0.00328	**
Fracture fluid	All	Genus	Unclassified Alphaproteobacteria	0.8400	1.0000	0.917	0.00652	**
Fracture fluid	All	Genus	*Erysipelothrix*	0.7652	1.0000	0.875	0.00388	**
Fracture fluid	All	Genus	Unclassified Acholeplasmataceae	0.7623	1.0000	0.873	0.01242	*
Fracture fluid	All	Genus	Unclassified TC1	0.7587	1.0000	0.871	0.01035	*
Fracture fluid	All	Genus	Unclassified Firmicutes	0.7306	1.0000	0.855	0.01000	**
Fracture fluid	All	Genus	Unclassified Bacteria	0.6956	1.0000	0.834	0.01128	*
Fracture fluid	All	Genus	Unclassified Gammaproteobacteria	0.6671	1.0000	0.817	0.02900	*
Fracture fluid	All	Genus	Unclassified Microbacteriaceae	0.6513	1.0000	0.807	0.00429	**
Fracture fluid	All	Genus	*Hydrogenophaga*	0.6443	1.0000	0.804	0.00328	**
Fracture fluid	All	Genus	*Legionella*	0.6281	1.0000	0.793	0.02357	*
Fracture fluid	All	Genus	Unclassified Desulfotomaculales Incertae Sedis	0.6050	1.0000	0.778	0.02703	*
Fracture fluid	All	Genus	Unclassified Thermotaleaceae	0.5498	1.0000	0.742	0.02919	*
Fracture fluid	All	Genus	*Roseococcus*	0.5445	1.0000	0.738	0.00932	**
Fracture fluid	All	Genus	Unclassified Rhizobiaceae	0.4737	1.0000	0.688	0.04481	*
Methane	All	Genus	Proteiniphilum	1.0000	0.8333	0.913	0.00043	***
Methanol	All	Genus	Unclassified Anaerolineae	1.0000	0.5	0.707	0.04	*
Microcosm	All	Genus	Unclassified Caulobacteraceae	1.0000	1.0000	1.0000	0.00328	**
Microcosm	All	Genus	*Desulfosporosinus*	0.9980	1.0000	0.999	0.00800	**
Microcosm	All	Genus	Unclassified Peptostreptococcales-Tissierellales	1.0000	'0.9583	0.979	0.02824	*
Microcosm	All	Genus	Unclassified Pseudomonadaceae	1.0000	0.8750	0.935	0.04123	*
Acetate	All	ASV	*Pseudomonas* (ASV_2)	0.501	1.0000	0.708	0.00009	***
Fracture fluid	All	ASV	Unclassified Comamonadaceae (ASV_101)	1.0000	1.0000	1.000	0.00328	**
Fracture fluid	All	ASV	*Silanimonas* (ASV_104)	1.0000	1.0000	1.000	0.00328	**
Fracture fluid	All	ASV	Unclassified Comamonadaceae (ASV_111)	1.0000	1.0000	1.000	0.00328	**
Fracture fluid	All	ASV	Unclassified Proteobacteria (ASV_112)	1.0000	1.0000	1.000	0.00328	**
Fracture fluid	All	ASV	Unclassified TC1 (ASV_118)	1.0000	1.0000	1.000	0.00328	**
Fracture fluid	All	ASV	Unclassified Bacteria (ASV_127)	1.0000	1.0000	1.000	0.00328	**
Fracture fluid	All	ASV	*Actinotalea* (ASV_142)	1.0000	1.0000	1.000	0.00328	**
Fracture fluid	All	ASV	*Erysipelothrix* (ASV_144)	1.0000	1.0000	1.000	0.00328	**
Fracture fluid	All	ASV	Unclassified Desulfotomaculales Incertae Sedis (ASV_154)	1.0000	1.0000	1.000	0.00328	**
Fracture fluid	All	ASV	*Sphingorhabdus* (ASV_163)	1.0000	1.0000	1.000	0.00328	**
Fracture fluid	All	ASV	Unclassified Microbacteriaceae (ASV_173)	1.0000	1.0000	1.000	0.00328	**
Fracture fluid	All	ASV	*Dethiosulfatibacter* (ASV_178)	1.0000	1.0000	1.000	0.00328	**
Fracture fluid	All	ASV	*Hydrogenophaga* (ASV_20)	1.0000	1.0000	1.000	0.00328	**
Fracture fluid	All	ASV	*Hydrogenophaga* (ASV_203)	1.0000	1.0000	1.000	0.00328	**
Fracture fluid	All	ASV	Unclassified Rhizobiaceae (ASV_217)	1.0000	1.0000	1.000	0.00328	**
Fracture fluid	All	ASV	*Hydrogenophaga* (ASV_22)	1.0000	1.0000	1.000	0.00328	**
Fracture fluid	All	ASV	Unclassified Acholeplasmataceae (ASV_23)	1.0000	1.0000	1.000	0.00328	**
Fracture fluid	All	ASV	*Seohaeicola* (ASV_24)	1.0000	1.0000	1.000	0.00328	**
Fracture fluid	All	ASV	Unclassified Thermotaleaceae (ASV_248)	1.0000	1.0000	1.000	0.00328	**
Fracture fluid	All	ASV	*Roseococcus* (ASV_249)	1.0000	1.0000	1.000	0.00328	**
Fracture fluid	All	ASV	*Erysipelothrix* (ASV_253)	1.0000	1.0000	1.000	0.00328	**
Fracture fluid	All	ASV	*Brevundimonas* (ASV_26)	1.0000	1.0000	1.000	0.00328	**
Fracture fluid	All	ASV	*Pseudorhodobacter* (ASV_27)	1.0000	1.0000	1.000	0.00328	**
Fracture fluid	All	ASV	*Brevundimonas* (ASV_31)	1.0000	1.0000	1.000	0.00328	**
Fracture fluid	All	ASV	*Hydrogenophaga* (ASV_4)	1.0000	1.0000	1.000	0.00328	**
Fracture fluid	All	ASV	*Pseudomonas* (ASV_45)	1.0000	1.0000	1.000	0.00328	**
Fracture fluid	All	ASV	*Brevundimonas* (ASV_65)	1.0000	1.0000	1.000	0.00328	**
Fracture fluid	All	ASV	Unclassified Comamonadaceae (ASV_78)	1.0000	1.0000	1.000	0.00328	**
Fracture fluid	All	ASV	*Phenylobacterium* (ASV_81)	1.0000	1.0000	1.000	0.00328	**
Fracture fluid	All	ASV	Unclassified Microbacteriaceae (ASV_86)	1.0000	1.0000	1.000	0.00328	**
Fracture fluid	All	ASV	Unclassified Desulfotomaculales Incertae Sedis (ASV_91)	1.0000	1.0000	1.000	0.00328	**
Fracture fluid	All	ASV	*Legionella* (ASV_97)	1.0000	1.0000	1.000	0.00328	**

Bacteria

Set	Dataset	Level	Taxa ID	A (specificity)	B (fidelity)	IndVal stat	p-value	significance
Methane	All	ASV	Unclassified Microbacteriaceae (ASV_110)	0.9218	0.8333	0.876	0.00039	***
Methane	All	ASV	Unclassified Proteobacteria (ASV_150)	0.8454	0.8333	0.839	0.00623	**
Methane	All	ASV	Unclassified Rhodobacteraceae (ASV_56)	0.6673	0.8333	0.746	0.01820	*
Methane	All	ASV	*Pseudorhodobacter* (ASV_8)	0.5977	1.0000	0.763	0.00577	**
Methane	All	ASV	*Proteiniphilum* (ASV_168)	1.0000	0.5000	0.707	0.04000	*
Methanol	All	ASV	*Hydrogenophaga* (ASV_6)	0.4703	1.0000	0.686	0.00327	**
Methanol	All	ASV	*Hydrogenophaga* (ASV_12)	0.4789	1.0000	0.692	0.00384	**
Methanol	All	ASV	Unclassified Rhodobacteraceae (ASV_96)	0.4891	1.0000	0.699	0.04639	*
Methanol	All	ASV	Unclassified Gammaproteobacteria (ASV_67)	0.4412	1.0000	0.664	0.01915	*
Methanol	All	ASV	Unclassified Comamonadaceae (ASV_16)	0.4110	1.0000	0.641	0.03875	*
Methanol	All	ASV	Unclassified Anaerolineae (ASV_113)	1.0000	0.5000	0.707	0.03920	*
Methanol	All	ASV	*Sphaerochaeta* (ASV_167)	1.0000	0.5000	0.707	0.04023	*
None	All	ASV	*Desulfosporosinus* (ASV_39)	0.9970	0.8333	0.911	0.0158	*
Methanol	Sessile	Genus	Unclassified Thermotaleaceae	0.8879	1.0000	0.942	0.0183	*
Methanol	Sessile	Genus	Unclassified Comamonadaceae	0.5463	1.0000	0.739	0.0155	*
Methanol	Sessile	Genus	EUB33-2	0.5296	1.0000	0.787	0.0183	*
Methanol	Sessile	Genus	*Brevundimonas*	0.4260	1.0000	0.653	0.0360	*
Methanol	Sessile	Genus	*Hydrogenophaga*	0.5283	1.0000	0.727	0.0183	*
Fracture fluid	Planktic	Genus	*Silanimonas*	1.0000	1.0000	1.000	0.0109	*
Fracture fluid	Planktic	Genus	*Leucobacter*	1.0000	1.0000	1.000	0.0109	*
Fracture fluid	Planktic	Genus	*Sphingorhabdus*	1.0000	1.0000	1.000	0.0109	*
Fracture fluid	Planktic	Genus	*Seohaeicola*	1.0000	1.0000	1.000	0.0109	*
Fracture fluid	Planktic	Genus	*Roseococcus*	1.0000	1.0000	1.000	0.0109	*
Fracture fluid	Planktic	Genus	Unclassified Burkholderiales	1.0000	1.0000	1.000	0.0109	*
Fracture fluid	Planktic	Genus	Unclassified Thermotaleaceae	1.0000	1.0000	0.959	0.0109	*
Fracture fluid	Planktic	Genus	Unclassified Desulfuromonadaceae	1.0000	1.0000	1.000	0.0109	*
Fracture fluid	Planktic	Genus	*Phenylobacterium*	1.0000	1.0000	1.000	0.0109	*
Fracture fluid	Planktic	Genus	Unclassified TC1	0.9887	1.0000	0.994	0.0218	*
Fracture fluid	Planktic	Genus	Unclassified SRB2	0.8964	1.0000	0.947	0.0293	*
Fracture fluid	Planktic	Genus	Unclassified Firmicutes	0.9675	1.0000	0.984	0.0220	*
Fracture fluid	Planktic	Genus	*Erysipelothrix*	0.9151	1.0000	0.957	0.0220	*
Fracture fluid	Planktic	Genus	Unclassified Acholeplasmataceae	0.8593	1.0000	0.923	0.0109	*
Fracture fluid	Planktic	Genus	*Hydrogenophaga*	0.7253	1.0000	0.852	0.0109	*
Fracture fluid	Planktic	Genus	Unclassified Alphaproteobacteria	0.9781	1.0000	0.989	0.0222	*
Fracture fluid	Planktic	Genus	Unclassified Rhizobiaceae	0.6720	1.0000	0.852	0.0495	*
Methane	Planktic	Genus	*Proteiniphilum*	1.0000	1.0000	1.000	0.011	*

Supplementary Table 10. Correlation indices for bacterial communities.

Bacteria						
Phase	Dataset	Level	Id	r.g. stat	p-value	significance
Fracture fluid	All	Genus	*Phenylobacterium*	0.999	0.0033	**
Fracture fluid	All	Genus	*Leucobacter*	0.998	0.0033	**
Fracture fluid	All	Genus	Unclassified Desulfuromonadaceae	0.983	0.0033	**
Fracture fluid	All	Genus	*Silanimonas*	0.975	0.0033	**
Fracture fluid	All	Genus	*Seohaeicola*	0.966	0.0033	**
Fracture fluid	All	Genus	*Hydrogenophaga*	0.962	0.0033	**
Fracture fluid	All	Genus	Unclassified Burkholderiales	0.850	0.0033	**
Fracture fluid	All	Genus	*Erysipelothrix*	0.921	0.0033	**
Fracture fluid	All	Genus	Unclassified Microbacteriaceae	0.740	0.0092	**
Fracture fluid	All	Genus	Unclassified Alphaproteobacteria	0.878	0.0033	**
Fracture fluid	All	Genus	Unclassified Firmicutes	0.817	0.009	**
Fracture fluid	All	Genus	Unclassified TC1	0.807	0.009	**
Fracture fluid	All	Genus	*Sphingorhabdus*	0.799	0.0033	**
Fracture fluid	All	Genus	Unclassified Bacteria	0.727	0.0124	**
Fracture fluid	All	Genus	*Legionella*	0.712	0.00973	**
Fracture fluid	All	Genus	Unclassified Gammaproteobacteria	0.666	0.02770	*
Fracture fluid	All	Genus	Unclassified Acholeplasmataceae	0.689	0.0127	*
Fracture fluid	All	Genus	Unclassified Desulfotomaculales Incertae Sedis	0.645	0.02443	*
Methane	All	Genus	*Proteiniphilum*	0.743	0.0117	*
Methane	All	Genus	*Pseudorhodobacter*	0.599	0.0292	*
Microcosms	All	Genus	*Pseudomonas*	0.779	0.0023	**
Microcosms	All	Genus	Unclassified Caulobacteraceae	0.671	0.0135	*
Acetate	All	ASV	*Pseudomonas* (ASV_2)	0.721	0.00958	**
Methane	All	ASV	Unclassified Microbacteriaceae (ASV_110)	0.820	0.00632	**
Methane	All	ASV	Unclassified Proteobacteria (ASV_150)	0.648	0.00937	**
Methane	All	ASV	Unclassified Rhodobacteraceae (ASV_56)	0.724	0.01237	*
Methane	All	ASV	*Pseudorhodobacter* (ASV_8)	0.683	0.01109	*
Methanol	All	ASV	*Hydrogenophaga* (ASV_6)	0.694	0.0114	*
Methanol	All	ASV	*Hydrogenophaga* (ASV_12)	0.671	0.0184	*
Methanol	All	ASV	*Sphaerochaeta* (ASV_167)	0.617	0.0402	*
Methanol	All	ASV	Unclassified Bacteria (ASV_230)	0.575	0.0377	*
Methanol	Sessile	Genus	*Hydrogenophaga*	0.926	0.0183	*
Methanol	Sessile	Genus	Unclassified Comamonadaceae	0.911	0.0183	*
Methanol	Sessile	Genus	EUB33-2	0.769	0.0177	*
Methanol	Sessile	Genus	Unclassified Thermotaleaceae	0.788	0.0183	*
Methanol	Sessile	Genus	*Brevundimonas*	0.754	0.0360	*
Methane	Planktic	Genus	*Proteiniphilum*	0.712	0.0217	*

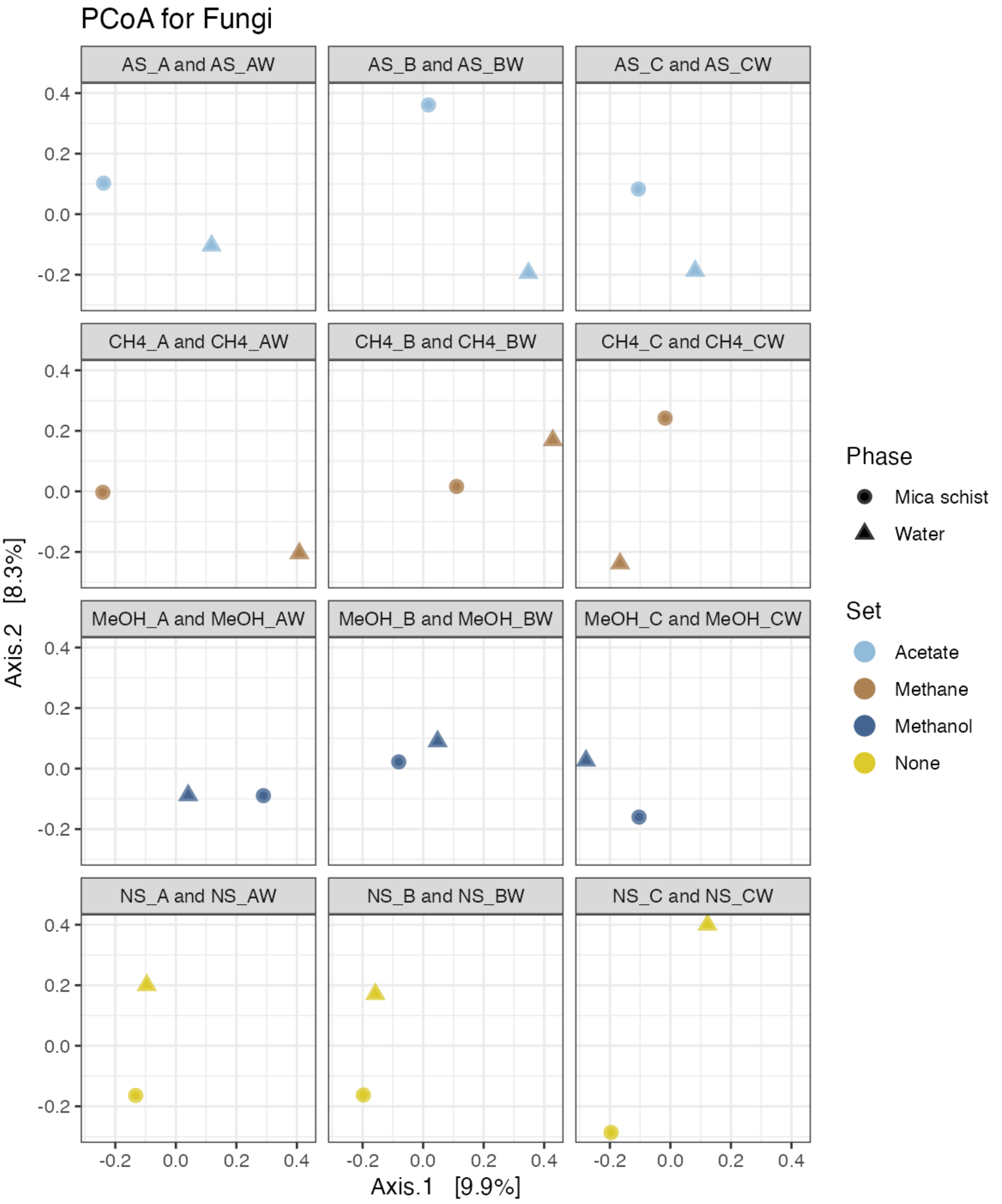

Supplementary Figure 4. The principal coordinate analysis for the fungal communities. Dissimilarities between different phases in different microcosms without substrate addition or supplied with acetate, methane, or methanol.

Supplementary Table 11. Alpha diversity measures for the fungal communities.

	Observed	Chao1	se.chao1	ACE	se.ACE	Shannon	Simpson
FF_A	4	4	0.0	4	1	0.7	0.4
FF_B	38	38	0.0	38	2	2.7	0.9
MeOH_A	23	26	4.2	27	3	2.1	0.8
MeOH_AW	18	18	0.0	18	2	1.2	0.5
MeOH_B	34	34	0.2	34	3	1.8	0.8
MeOH_BW	34	34	0.0	34	3	2.4	0.8
MeOH_C	20	20	0.0	20	2	0.9	0.3
MeOH_CW	38	38	0.0	38	3	2.6	0.8
NS_A	23	23	0.0	23	2	1.0	0.4
NS_AW	22	22	0.0	22	2	1.6	0.6
NS_B	28	28	1.3	29	3	1.9	0.7
NS_BW	15	16	2.3	16	2	1.4	0.6
NS_C	25	25	0.5	25	2	1.1	0.5
NS_CW	33	39	7.3	36	3	2.2	0.8
CH4_A	33	33	0.5	33	3	2.1	0.8
CH4_AW	13	13	0.2	13	2	2.1	0.8
CH4_B	16	16	0.5	16	2	0.6	0.2
CH4_BW	18	18	0.5	18	2	1.4	0.7
CH4_C	19	20	2.3	21	2	1.8	0.8
CH4_CW	25	25	0.2	25	2	1.8	0.8
AS_A	27	27	0.0	27	2	1.9	0.7
AS_AW	20	20	1.3	21	2	1.8	0.7
AS_B	24	24	0.0	24	2	2.2	0.8
AS_BW	19	20	2.3	20	2	1.4	0.6
AS_C	22	22	0.0	22	2	2.7	0.9
AS_CW	25	25	0.1	25	3	2.3	0.9

Supplementary Table 12. Indicator fungi in different treatments, Indicator Values and their significance

Fungi								
Set	Dataset	Level	Id	A (specificity)	B (fidelity)	IndVal stat	p-value	significance
Acetate	All	Genus	*Sarocladium*	0.7734	1.0000	0.879	0.00877	**
Acetate	All	Genus	Unclassified Didymellaceae	0.9128	0.6667	0.780	0.04923	*
Fracture fluid	All	Genus	*Fusarium*	1.0	1.0	1.0	0.00328	**
Fracture fluid	All	Genus	*Meyerozyma*	1.0	1.0	1.0	0.00328	**
Methanol	All	Genus	*Naganishia*	0.9704	1.0000	0.985	0.00307	**
Methanol	All	Genus	Unclassified Diatrypaceae	0.9768	0.8333	0.902	0.01900	**
Methanol	All	Genus	*Bartalinia*	0.9489	0.8333	0.889	0.02144	*
Methanol	All	Genus	Unclassified Clavulinaceae	0.9704	0.6667	0.804	0.02027	*
Methanol	All	Genus	*Humicola*	1.0000	0.5000	0.707	0.03683	*
Acetate	All	ASV	*Dissoconium* (ASV_44)	0.9597	0.6667	0.800	0.0458	*
Acetate	All	ASV	Unclassified Didymellaceae (ASV_16)	0.9583	0.6667	0.799	0.0463	*
Methanol	All	ASV	Unclassified Diatrypaceae (ASV_2)	0.9931	0.8333	0.910	0.01889	*
Methanol	All	ASV	*Aspergillus* (ASV_17)	0.9893	0.8333	0.908	0.01643	*
Methanol	All	ASV	*Bartalinia* (ASV_67)	0.9622	0.8333	0.895	0.02089	*
Methanol	All	ASV	*Phaeotremella* (ASV_113)	0.8852	0.8333	0.859	0.00369	**
Methanol	All	ASV	*Malassezia* (ASV_111)	0.8438	0.8333	0.839	0.01329	*
Methanol	All	ASV	Unclassified Clavulinaceae (ASV_27)	0.9896	0.6667	0.812	0.03101	*
Methanol	All	ASV	*Naganishia* (ASV_42)	0.9864	0.6667	0.811	0.01651	*
Methanol	All	ASV	*Penicillium* (ASV_72)	0.9034	0.6667	0.776	0.04494	*
Methanol	All	ASV	*Trichosporon* (ASV_101)	0.6707	0.8333	0.748	0.03249	*
Methanol	All	ASV	*Humicola* (ASV_110)	1.0000	0.5000	0.707	0.03767	*
Fracture fluid	Planktic	Genus	*Fusarium*	1.0	1.0	1.0	0.0109	*
Fracture fluid	Planktic	Genus	*Meyerozyma*	1.0	1.0	1.0	0.0109	*
Acetate	Planktic	Genus	*Sarocladium*	0.8983	1.0000	0.948	0.0294	*
Methane	Planktic	Genus	Unclassified Leotiomycetes	0.8473	1.0000	0.92	0.026	*
Methanol	Planktic	Genus	*Naganishia*	0.9870	1.0000	0.993	0.00689	**
Methanol	Planktic	Genus	Unclassified Diatrypaceae	0.9728	1.0000	0.986	0.00876	**
Methanol	Planktic	Genus	Unclassified Clavulinaceae	0.9282	1.0000	0.963	0.00876	**
Methanol	Planktic	Genus	*Trichosporon*	0.8494	1.0000	0.922	0.02878	*
None	Planktic	Genus	*Vexillomyces*	0.7657	1.0000	0.875	0.0175	*
Acetate	Sessile	Genus	*Vexillomyces*	0.9849	1.0000	0.992	0.0184	*

Supplementary Table 13. Indicator fungi in different treatments, correlation indices and their significance

Fungi						
Set	Dataset	Level	Id	r.g. stat	p-value	significance
Fracture fluid	All	Genus	*Fusarium*	0.795	0.00328	*
Fracture fluid	All	Genus	*Meyerozyma*	0.670	0.00328	*
Acetate	All	Genus	*Sarocladium*	0.611	0.039	*
Methanol	All	ASV	*Phaeotremella* (ASV_113)	0.769	0.00657	**
Methanol	All	ASV	*Malassezia* (ASV_111)	0.684	0.01322	*
Methanol	All	ASV	*Naganishia* (ASV_90)	0.608	0.04502	*
Fracture fluid	Planktic	Genus	*Fusarium*	0.795	0.0109	*
Fracture fluid	Planktic	Genus	*Meyerozyma*	0.670	0.0109	*
Acetate	Planktic	Genus	*Sarocladium*	0.611	0.0368	*
Methanol	Planktic	Genus	Unclassified Clavulinaceae	0.922	0.0193	*
Methanol	Planktic	Genus	Unclassified Diatrypaceae	0.895	0.0200	*
Methanol	Planktic	Genus	*Trichosporon*	0.715	0.0433	*
None	Planktic	Genus	*Vexillomyces*	0.743	0.0394	*
Acetate	Sessile	Genus	*Vexillomyces*	0.565	0.0181	*

Supplementary Table 14. The pH and DNA concentration (ng/ µL) across samples. Kietäväinen (2017) reported several pH measurements from the depth of 500 m fracture fluids for October 2010.

Sample ID	pH	DNA ng/µL	Reference
FF_A	6.3 - 8.7	n.a.	Kietäväinen, 2017
FF_B	6.3 - 8.7	n.a.	Kietäväinen, 2017
NS_A	measured from water phase	3.7	-
NS_B	measured from water phase	n.d.	-
NS_C	measured from water phase	n.d.	-
MeOH_A	measured from water phase	2.8	-
MeOH_B	measured from water phase	5.0	-
MeOH_C	measured from water phase	2.9	-
CH4_A	measured from water phase	0.3	-
CH4_B	measured from water phase	1.1	-
CH4_C	measured from water phase	1.4	-
AS_A	measured from water phase	1.6	-
AS_B	measured from water phase	3.2	-
AS_C	measured from water phase	4.2	-
NS_AW	5.5	3.3	-
NS_BW	5.7	2.7	-
NS_CW	6.2	1.7	-
MeOH_AW	6.65	3.8	-
MeOH_BW	6.86	3.4	-
MeOH_CW	6.81	4.4	-
CH4_AW	6.62	1.3	-
CH4_BW	6.56	2.5	-
CH4_CW	6.57	1.7	-
AS_AW	6.42	n.d.	-
AS_BW	6.56	0.4	-
AS_CW	6.53	1.7	-

Abbreviations in the table: n.a. = not analyzed, n.d. = not detected

References

Fullerton, et al., 2021. Effect of tectonic processes on biosphere–geosphere feedbacks across a convergent margin. Nat. Geosci.

Kietäväinen, R. 2017. Deep Groundwater Evolution at Outokumpu, Eastern Finland: From Meteoric Water to Saline Gas-Rich Fluid.

Salter et al., 2014.Reagent and laboratory contamination can critically impact sequence-based microbiome analyses. BMC Biology 12:87.

Sheik et al., 2018. Identification and Removal of Contaminant Sequences From Ribosomal Gene Databases: Lessons From the Census of Deep Life. Front. Microbiol. 9:840.
